# Bone regeneration during osteoporosis: a translational *in vivo* monitoring of callus mechanical parameters

**DOI:** 10.3389/fbioe.2025.1646500

**Published:** 2025-10-29

**Authors:** Juan J. Toscano-Angulo, Juan Mora-Macías, Pablo Blázquez-Carmona, Manuel Sánchez-Raya, Juan Morgaz, Juan Antonio Gómez-Galán, M. M. Granados, Jaime Domínguez, Esther Reina-Romo

**Affiliations:** ^1^ Departamento de Ingeniería Mecánica y Fabricación, Escuela Técnica Superior de Ingeniería, Universidad de Sevilla, Sevilla, Spain; ^2^ Departamento de Ingeniería Minera, Mecánica, Energética y de la Construcción, Escuela Técnica Superior de Ingeniería, Universidad de Huelva, Huelva, Spain; ^3^ Departamento de Ingeniería Mecánica y Diseño Industrial, Escuela Superior de Ingeniería, Universidad de Cádiz, Puerto Real, Spain; ^4^ Departamento de Ingeniería Electrónica, de Sistemas Informáticos y Automática, Escuela Técnica Superior de Ingeniería, Universidad de Huelva, Huelva, Spain; ^5^ Departamento de Medicina y Cirugía Animal, Facultad de Veterinaria, Universidad de Córdoba, Córdoba, Spain

**Keywords:** osteoporosis, bone regeneration, bone transport, distraction osteogenesis, distraction force, viscoelastic model, gait analysis, x-ray

## Abstract

**Introduction:**

Regenerating osteoporotic bone remains challenging due to healing complications such as non-unions. Distraction osteogenesis is a promising technique for bone repair, but its efficacy under osteoporotic conditions is poorly understood. This study provides *in vivo* quantitative translational knowledge on the influence of osteoporosis in distraction callus mechanics.

**Methods:**

Fifteen Merino sheep were induced with osteoporosis. A 15 mm bone defect in the right hind metatarsus was treated with distraction osteogenesis and stabilized with an instrumented external fixator. Callus distraction forces and relaxation were recorded and modeled to assess the viscoelastic behavior of the organic matrix. Callus ossification and mechanics were assessed using x-ray imaging and gait analysis. The results were compared to those from similar studies involving non-osteoporotic subjects.

**Results:**

Osteoporosis significantly reduced distraction force peaks and relaxation (*p* < 0.05), with effects diminishing over the relaxation time. The elastic component of the organic matrix, especially ground substance, was significantly impaired (*p* < 0.05). X-ray follow-up and gait analysis revealed that half of the pathologic animals recovered comparably to non-osteoporotic subjects, while the others exhibit lateralized mineralization and reduced load bearing capacity.

**Discussion:**

Osteoporosis led to a 50% reduction in the viscoelastic response of the distraction callus, likely due to an impaired osteoblastic matrix synthesis. Furthermore, osteoporotic patients undergoing distraction osteogenesis for critical-size defects may experience delayed consolidation with heterogeneous mineralization, which may be linked to early deficits in organic matrix formation.

## 1 Introduction

Osteoporosis is a chronic widespread metabolic disorder characterized by reduced bone mineral density and microstructural impairment, predisposing patients to a higher fracture risk. Secondary osteoporosis is the underlying effect of several diseases and their pharmacological treatments, disrupting the balance between bone formation and resorption (Ahmed and Elmantaser, 2009). Common anti-inflammatory drugs such as glucocorticoids induce osteocyte apoptosis, thus increasing the bone fragility (Whittier and Saag, 2016). The consequences are low-energy fractures, commonly referred to as fragility fractures. These fractures are associated with a mortality rate of up to 33% within the first year (Guzon-Illescas et al., 2019), along with a high probability of irreversible disability and long-term dependency (Lems and Raterman, 2017; Bae et al., 2019). Almost 12,000 fragility fractures occur per day in Europe, representing a cost of €56.9 billion annually to the healthcare system (Kanis et al., 2021).

The fracture healing process has been frequently studied in osteoporotic rodent models (Kubo et al., 1999; Namkung-Matthai et al., 2001; Xu et al., 2004; Yingjie et al., 2007; McCann et al., 2008; Li et al., 2010; Chow et al., 2011; Oliver et al., 2013; Chung et al., 2014; Kido et al., 2014; Thormann et al., 2014; Wehrle et al., 2015; Fischer et al., 2017; Fischer et al., 2018; Wong et al., 2019). By using different approaches and measurement techniques (x-ray follow-up, histology, histomorphology, serum biomarkers or biomechanical testing, among others), these studies agree that osteoporosis impairs fracture healing capability. The resulting osteoporotic woven tissue reported lower bone mineral density, fracture load and osteoclastic activity compared to non-osteoporotic woven bone. These findings align with the scarce available literature on fracture healing in osteoporotic large animal models (Lill et al., 2003; Bindl et al., 2013). In these challenging bone regeneration scenarios caused by osteoporosis, the primary concern is the potential for delayed healing, non-unions, bone deformities, chronic pain, and post-surgical complications, including infections (Shih et al., 2022; Hu et al., 2023; Leslie and Baumgaertner, 2023).

Introduced by Ilizarov (Ilizarov, 1989a; Ilizarov, 1989b), distraction osteogenesis (DO) is a clinically relevant bone regeneration technique. It involves the controlled gradual separation, known as distraction, of two bone fragments following an osteotomy, promoting the formation of new bone tissue by means of bone mechanotransduction (Compton et al., 2015; Runyan and Gabrick, 2017; Adejuyigbe et al., 2024). Based on this principle, bone transport has become a gold-standard orthopedic technique for reconstructing of critical-size bone defects caused by traumas, infections, tumors or prior surgical interventions (Brunner et al., 1994; Claes et al., 2000; Rozbruch et al., 2008; Li et al., 2023). This clinical procedure enables gradual distraction via a bone fragment positioned within a larger bone gap, leading to double ossification. Nevertheless, despite the need to apply DO in osteoporotic bone regeneration and the high prevalence of fragility fractures in elderly patients (Kanis et al., 2000; Curtis et al., 2016; Wu et al., 2021), there is extremely limited literature on osteoporotic DO. In this context, some experimental studies have explored the effect of osteoporosis on DO using small animal models (Arslan et al., 2003; Tatehara et al., 2011; Sun et al., 2014). For instance, Arslan et al. (2003) found that osteoporosis negatively impacts mandibular DO outcomes, with delayed callus formation and remodeling observed in ovariectomized rabbit models. Similarly, Tatehara et al. (2011) reported these findings by concluding reduced femoral woven bone volume and increased osteoclastic activity in an osteoporotic rat model. Finally, Sun et al. (2014) further demonstrated the potential of gene therapy to promote the formation of DO woven tissue in osteoporotic rabbit’s mandible.

Although all these works provided relevant contributions to the study of osteoporotic DO, they present several limitations. In this regard, the findings from small animal models should be interpreted with caution. This is due to the biological differences between these models and humans, including discrepancies in bone regeneration rates, responses to treatment, mechanical loading, and the overall complexity of human osteoporosis (Turner, 2001; Lelovas et al., 2008). In contrast, large animal models are more suitable for osteoporosis research due to their greater physiological similarity to human and disease mechanism (Reinwald and Burr, 2008; Dias et al., 2018). In particular, ovariectomized sheep with glucocorticoid treatment model shows consistent reductions in bone mineral density, volume fraction and strength in trabecular tissue (Lill et al., 2002; Augat et al., 2003; Zarrinkalam et al., 2009; Ding et al., 2010; Oheim et al., 2012; Toscano-Angulo et al., 2025), similar to what happens in postmenopausal women with secondary osteoporosis (Dias et al., 2018). As far as the authors know, only Toscano-Angulo et al. (2025) have addressed the complexity of DO in this osteoporotic large animal model. They provided insights into the skeletal quality alterations produced by DO in osteoporotic animals in different bone tissues and locations. Another remarkable knowledge gap is the lack of quantitative information about DO woven maturation in osteoporotic subjects by using *in vivo* analysis techniques. An additional gap of knowledge is the lack of quantitative information about the osteoporotic distraction callus maturation obtained through *in vivo* analysis techniques. In this regard, previous experimental studies conducted in non-osteoporotic large animal models revealed that continuous *in vivo* monitoring from early stages of the callus mechanical parameters has a valuable direct relationship with the ossification process (Brunner et al., 1994; Claes et al., 1995; 2000; Wee et al., 2011; Mora-Macías et al., 2015a; Mora-Macías et al., 2015b; Mora-Macías et al., 2016; Blázquez-Carmona et al., 2021a; Blázquez-Carmona et al., 2021b; Blázquez-Carmona et al., 2023a; Blázquez-Carmona et al., 2023b). Applied to osteoporotic subjects undergoing regeneration through DO, these analyses may help elucidate the mechanobiological differences induced by the disease. This knowledge would be highly relevant for assessing the feasibility of translating the DO process to clinical practice in fractured osteoporotic patients. Furthermore, the data would help in the development of *in silico* models to characterize and forecast the mechanical evolution of the osteoporotic distraction callus. However, as far as the authors know, this clinically meaningful information has not been previously explored.

*In vivo* DO studies have generally focused on two fundamental bone regeneration phases: the distraction phase, involving naïve and premineralized soft tissue, and the consolidation phase, during which mineralization occurs. The early premineralized stage of the distraction callus has been evaluated in several studies in non-osteoporotic sheep (Claes et al., 1995; 2000; Wee et al., 2011; Mora-Macías et al., 2016; Blázquez-Carmona et al., 2021b). The displacement of the bone fragment generates a callus traction force, which can be quantified through an instrumented external fixator. In bone lengthening contexts, Wee et al. (2011) found that increasing the daily distraction length is effective in reducing high distraction forces, which are related to increased callus stiffness and mineralization. On the other hand, Mora-Macías et al. (2016) developed two mathematical models to reproduce the callus mechanical behavior during bone transport. They suggested that at least 78% of the distraction force during bone lengthening comes from the elongation of the surrounding soft tissues, rather than the callus tissue itself. This finding was confirmed by Blázquez-Carmona et al. (2021b), who provided a viscoelastic model that reproduces the mechanical behavior of the surrounding soft tissue and the premineralized callus in bone lengthening. They concluded that surrounding soft tissue could notably increase the forces during the first minutes of distraction, with similar tissue relaxation in both DO processes. All these works provided relevant information on the influence of distraction forces on the maturation and ossification of DO woven tissue. However, as far as the authors know, the effect of osteoporosis on the mechanical properties of the distraction callus is still unknown. Furthermore, viscoelastic models of this premineralized tissue based on distraction forces offer the advantage of being able to mechanically differentiate between the main compounds of the organic matrix, synthesized by osteoblasts. This relevant differentiation applied to osteoporotic subjects would allow to estimate how the disease influences the distraction callus at the bone cellular activity level.

During the consolidation of the woven bone tissue, one of the critical questions for clinicians is how long the distracted callus needs to mature before the fixator can be safely removed. This issue is particularly sensitive in osteoporotic patients who are prone to non-union or delayed healing time (Nikolaou et al., 2009; Gorter et al., 2021). Traditional clinical x-ray follow-up and manual examination have proven to provide very limited and inaccurate information (Webb et al., 1996; Fisher et al., 2019). This lack of quantitative knowledge can be covered by performing gait analyses. From these non-invasive tests, *in vivo* mechanical parameters of the woven mineralization can be estimated, such as its load-bearing capacity and longitudinal stiffness (Morasiewicz et al., 2010; Mora-Macías et al., 2015a; Macías et al., 2015b; Blázquez-Carmona et al., 2021a; Blázquez-Carmona et al., 2023b; Blázquez-Carmona et al., 2023a; Pawik et al., 2021). In a non-osteoporotic sheep metatarsus model, Mora-Macías et al. (2015a) revealed that the distraction callus bearing capacity reached 80%–90% at 70 days after the bone surgery. At that time, they concluded that this non-osteoporotic immature tissue stiffens exponentially reaching up to 5.4–11.4 kN/mm. Performing similar analyses in osteoporotic conditions, using the same model and metatarsal DO protocol than Mora-Macías et al. (2015a), would allow for a quantitative comparison of callus mechanical properties, enabling estimation of fixation duration and clinical feasibility and translatability of DO for osteoporotic patients.

**FIGURE 1:**
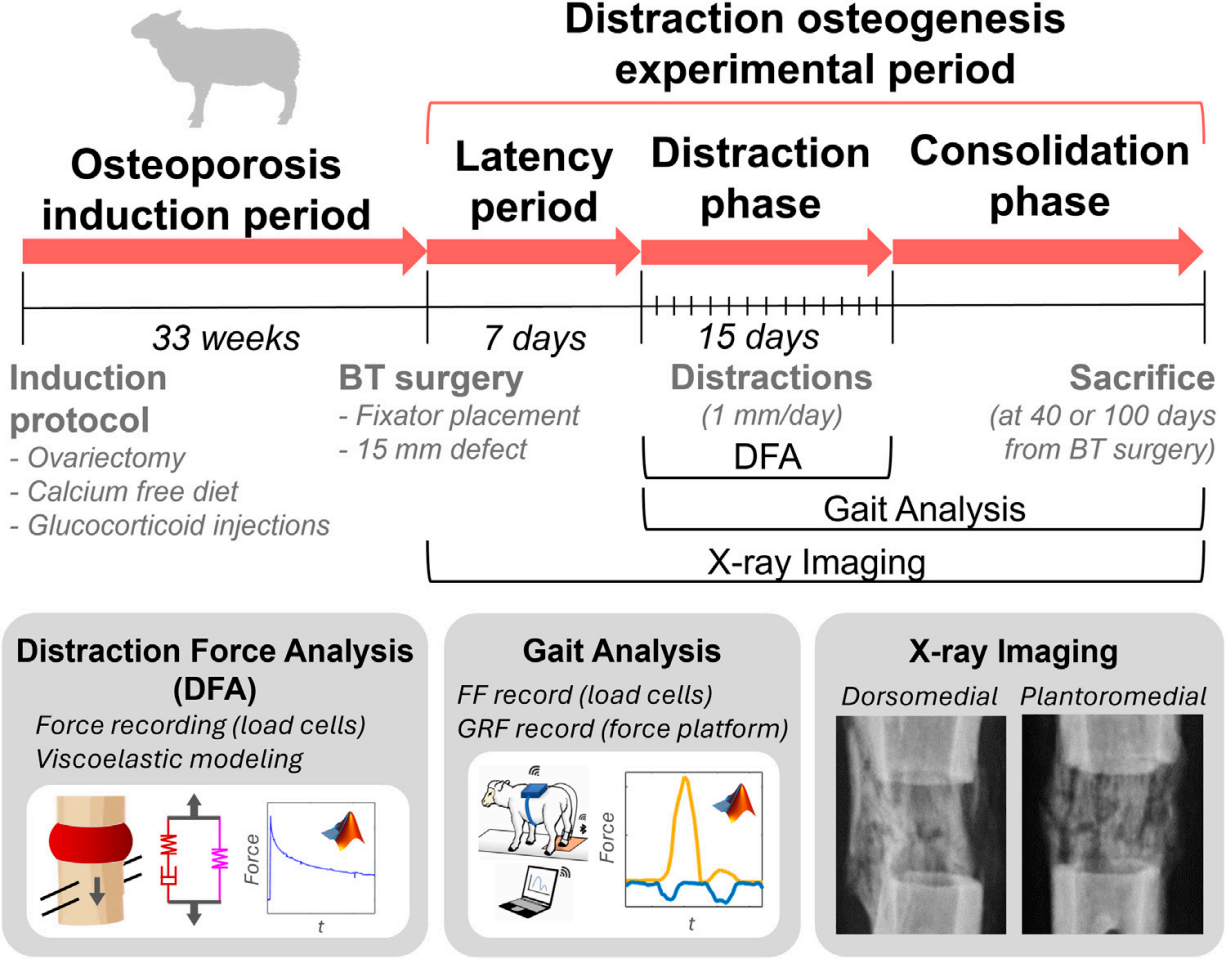
Study design overview: animal experimental periods, procedures and analysis performed.

The aim of this work is to *in vivo* quantitatively monitor bone regeneration during the process of DO in osteoporotic sheep. Distraction forces, relaxation and viscoelastic behavior of the early-stage premineralized distraction callus will be evaluated, showing the influence of osteoporosis on its mechanical behavior during the distraction phase (Figure 1). Moreover, the callus ossification will be assessed by measuring woven bone mechanical parameters (e.g., callus force and stiffness) in gait analyses supported by x-ray follow-up, revealing how osteoporosis affects mineral progression during the consolidation phase. The data will be compared with a non-osteoporotic control group (Mora-Macías et al., 2015a; Mora-Macías et al., 2015b; Mora-Macías et al., 2016; Blázquez-Carmona et al., 2021b) in order to isolate and elucidate the effect of the disease during the bone regeneration process. All these highly valuable translational findings will allow to overcome the lack of quantitative *in vivo* mechanical knowledge on osteoporotic bone regeneration using DO.

## 2 Materials and methods

### 2.1 Osteoporotic animal model and distraction osteogenesis protocol

Osteoporosis was experimentally induced in fifteen female Merino sheep, aged 2–4 years and weighing 60.2 ± 5.6 kg. Animal welfare was preserved throughout experimental periods in accordance with the ARRIVE guidelines, European (63/2010/EU) and national (RD 53/2013) regulations on preclinical research. The study received approval from the Ethics Committee of the University of Córdoba (Protocol Number: 2021PI/21). The subjects were randomly sourced from a research farm and identified by wool marking to avoid potential confounding factors. The specimens were in good health and had received standard vaccinations and deworming treatments. The minimum sample size was determined according to the variability reported in previous studies (Mora-Macías et al., 2015a; Mora-Macías et al., 2015b; Mora-Macías et al., 2016; Blázquez-Carmona et al., 2021a; Blázquez-Carmona et al., 2021b). All experimental procedures were conducted at the Clinical Veterinary Hospital of the University of Cordoba, where the sheep were housed in spacious, partially roofed and fenced outdoor areas.

Following the guidelines described by Zarrinkalam et al. (2009), osteoporosis induction involved firstly a bilateral ovariectomy. During the surgical intervention, the animal was under general anesthesia and intubated with continuous monitoring of body temperature, blood pressure, O_2_ saturation, exhaled CO_2_ levels, and electrocardiograms. Subsequently, the ovariectomized sheep were administered intramuscular glucocorticoid injections (500 mg Solu-Moderín^®^ diluted in 7.8 mL injectable water) every 3 weeks, along with a calcium-free diet (12% crude protein, 9% crude fiber, 6.5% crude ash, 2% crude fat, 0% calcium, 0.1% phosphorus and 0.1% sodium) till week 33 ± 2.5 after ovariectomy, when distraction osteogenesis experiments began (Figure 1). At this point, glucocorticoid administration was stopped to preserve postsurgical animal welfare. This osteoporotic induction protocol was successfully verified by reporting a mean reduction in trabecular bone mineral density of 6.4% in a previous study (Toscano-Angulo et al., 2025).

At 33 ± 2.5 weeks post-ovariectomy, a second surgical procedure was performed to generate a critical size-bone defect in the right hind metatarsus of the sheep. This defect was previously stabilized with implantation of an instrumented Ilizarov-type external fixator-distractor. The bone surgery was performed under the same anesthetic and physiological monitoring conditions as the ovariectomy. After the fixator placement, three separated diaphyseal osteotomies were performed using a guided oscillating saw which resulted in two bone segments (Toscano-Angulo et al., 2025). The proximal bone segment (25 mm length) was previously fixed to the distractor, whilst the distal bone segment was removed to create the critical-size defect (15 mm gap). After a 7-day latency period, the fixed bone fragment was distally displaced 1 mm/day (distraction) to fill the bone gap throughout a 15-day distraction phase (Figure 1). The DO protocol creates an elongation of the proximal osteotomy (distraction callus) and a compression of the critical-size defect (docking site callus). The woven tissue formed within the distraction callus was studied in a subsequent consolidation phase. The sheep were randomly slaughtered by an overdose of sodium pentobarbital IV Euthasol^®^ on day 40 or 100 after the bone surgery for an *ex vivo* study out of the scope of this work.

### 2.2 *In vivo* instrumentation and bone-fixator spring model

**FIGURE 2:**
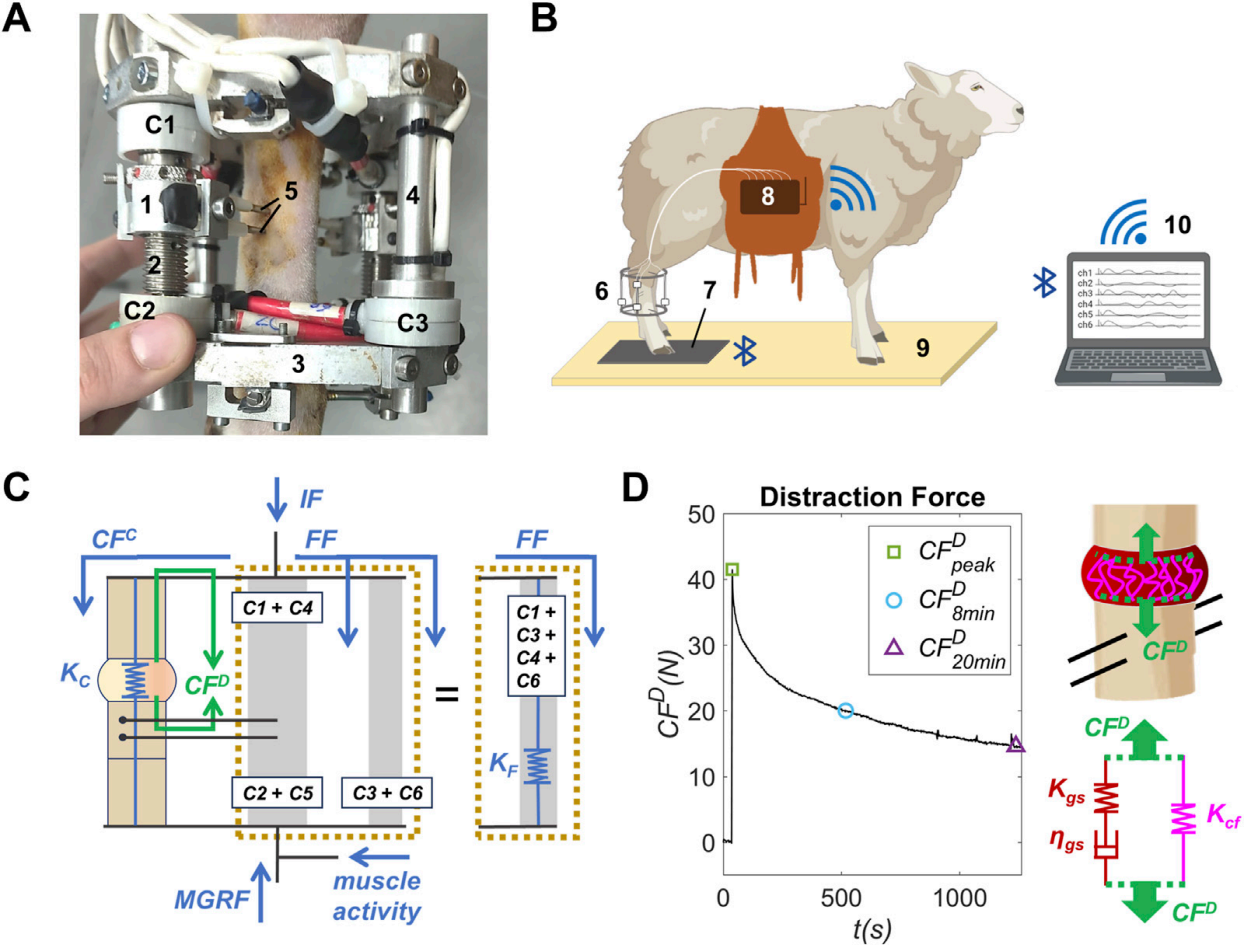
**(A)** Instrumented external fixator-distractor placed in the right hind metatarsus of an osteoporotic sheep: (1) distractor, (2) distraction bar, (3) stainless fixator ring frame, (4) simple bar, (5) Ø2.5 mm Steinmann pins, (C1) proximal load cell of the lateral distraction bar, (C2) distal load cell of the lateral distraction bar and (C3) load cell of the dorsal simple bar. **(B)** Instrumentation and facilities for gait analysis: (6) instrumented external fixator-distractor, (7) force platform, (8) data acquisition system, (9) gait corridor, (10) computer. **(C)** Bone-fixator spring model of the operated limb: forces and stiffness during gait analysis in blue and during distraction force test in green. The bars are illustrated in pairs for visual clarity. **(D)** Example of a callus distraction force (*CF* ^*D*^) measurement performed in an osteoporotic sheep, scheme of the premineralized distraction callus during a distraction performance, and viscoelastic spring-dumper model of premineralized distraction callus during the distraction performance.

The instrumentation used for *in vivo* monitoring of distraction force tests and gait analyses is presented in Figure 2. These analyses will be described in detail in the subsequent sections. The instrumented external fixator (Mora-Macías et al., 2015a; Mora-Macías et al., 2015b) employed consists of two stainless steel ring frames secured to the bone by means of inserted Ø4 mm Schanz screws. Four connecting bars (two distraction bars and two simple bars) instrumented with Burster^®^ 8431–6001 (Burster, Gernsbach, Germany) load cells (load range, ±1 kN; accuracy, ±1.5 N; diameter, 25.4 mm; height, 14 mm; mass, 40 g) link the rings and mechanically stabilize the bone gap surgically created. The fixed bone segment is attached to the lateral and medial distraction bars by means of Ø2.5 mm Steinmann pins. These 2 bars are designed with a threaded mechanical system (distractor) that enables a controlled longitudinal movement of the bone segment. As shown in Figure 2A, each distraction bar included two load cells between the rings and separated by the distractor (C1 proximal and C2 distal in the lateral bar, C4 proximal and C5 distal in the medial bar) to isolate the load distribution between the distraction and docking site calluses. Meanwhile, the simple bars are equipped with a load cell (C3 in the dorsal bar and C6 in the plantar bar).

As illustrated in Figure 2B, the load cells data were collected in real-time and transmitted via Wi-Fi to a computer using a custom developed data acquisition system (dimensions: 19 × 12 × 3.5 cm, weight 1 kg, sampling rate 50 Hz). A complete technical description of this device is provided in the Supplementary Material (Supplementary Material 1). The force measurement system was validated using the guidance of a previous study (Blázquez-Carmona et al., 2020). In gait analysis, the device was placed in a saddlebag worn by the animal. In addition, a Pasco^®^ PS-2141 (Pasco, Roseville, CA, EE. UU.) force platform (force measurement range, −1,100 to 4,400 N; sampling rate, 1,000 Hz; resolution, 0.1 N; dimensions, 35 × 35 cm; mass, 4 kg) was placed and securely fixed to a gait corridor to minimize vibration artifacts. This device monitored the force data of the sheep’s footsteps in real time and transmitted the recordings to a computer via Bluetooth.

All these force sensors allow the measurement of the callus distraction force (*CF* ^*D*^) in distraction force tests performed during the distraction phase, and the fixator force (*FF*) in gait analyses during the consolidation phase. According to the bone-fixator spring model illustrated in Figure 2C, *CF* ^*D*^ (green arrows) is the traction force experienced by the premineralized distraction callus after each daily distraction performance. It can be calculated from the load cell measurements using Equation 1 (Mora-Macías et al., 2016).

$${CF}^{D}=\left(C2+C5\right)-\left(C1+C4\right)$$ (1)

where *C1* and *C2* are the forces measured by the load cells C1 and C2 (proximal and distal, respectively) of the lateral distraction bar, and *C4* and *C5* are the forces measured by the load cells C4 and C5 (proximal and distal, respectively) of the medial distraction bar (Figures 2A,C).

Regarding gait analyses, *FF* is the maximum force through the fixator due to the sheep steps (blue arrows, Figure 2C). It is measured according to Equation 2 (Mora-Macías et al., 2015a).

FF=C1+C3+C4+C6 (2)

where *C3* is the force measured in the load cell C3 of the dorsal simple bar, and *C6* is the force measured in the load cell C6 of the plantar simple bar (Figures 2A,C).

The internal force (*IF*, Figure 2C) represents the total force transferred through the limb’s musculoskeletal system due to the maximum ground reaction force (*MGRF*) and muscle activity (Blázquez-Carmona et al., 2021a). In the operated limb, the internal force *IF* is distributed between the external fixator (fixator force, *FF*) and the bone callus (callus force or callus bearing capacity, *CF* ^*C*^) depending on its longitudinal stiffness (Figure 2C; Equation 3). Note that the mathematical expression of the callus distraction force during the distraction phase (*CF* ^*D*^, Equation 1) allows deleting the effect of the *IF* in force measurements.

$$IF=FF+{CF}^{C}$$ (3)

While the stiffness of the fixator is constant over time (*K*_*F*_ = 655 N/mm), the stiffness of the bone callus (*K*_*C*_) evolves throughout the consolidation phase due to its mineral formation. The callus bearing capacity could be neglected (*CF* ^*C*^ ≈ 0) during the first days after the bone surgery due to the lack of a callus mineral bridging connecting the remaining bone fragments. This hypothesis was established in accordance with x-ray follow-up. Thus, the *IF* is supported approximately in its totality by the fixator (*IF* ≈ *FF*) during the distraction phase. Furthermore, the *IF*/*MGRF* ratio was observed to remain approximately constant during this early regeneration phase according to previous studies (Dwyer et al., 1996; Mora-Macías et al., 2015b; Blázquez-Carmona et al., 2021a). In this sense, the *CF* ^*C*^ could be indirectly obtained as a function of *FF* and *MGRF* during the consolidation phase (Equation 4).

$${CF}^{C}=IF-FF=MGRF\cdot {\left(\bar{\frac{FF}{MGRF}}\right)}^{*}-FF$$ (4)

where mean (*FF*/*MGRF*)* is the mean value of the *FF*/*MGRF* ratio of early gait analyses in which there is no bridged callus.

Finally, the distraction callus longitudinal stiffness *K*_*C*_ during the consolidation phase could be estimated as presented in Equation 5.

$$K_{C}=\left(\frac{{CF}^{C}}{FF}\right)\cdot K_{F}$$ (5)

Considering that the longitudinal stiffness of the adjacent cortical fragments was infinite compared to that of the woven bone tissue, the stiffness of the metatarsus was dominated by the stiffness of the distraction callus.

### 2.3 Distraction force tests

A daily monitoring of the load cells was performed during the 15 days of distraction phase for each animal. During the distraction force test, the sheep was positioned in right lateral decubitus on the ground and immobilized without sedation to preserve animal welfare. The operated limb was kept elevated and supported in a sling to avoid confounders factors from the bodyweight or the load bearing of the limb.

The callus distraction force *CF* ^*D*^ was indirectly estimated in each test from the load cells measurements (Equation 1). A *CF* ^*D*^ recording example is presented in Figure 2D. Load cells measurements began before performing the distraction and continued for up to 20 min post-distraction. In this sense, a force peak was reported at the time of distraction performance, followed by a post-distraction relaxation of the premineralized distraction callus due to its viscoelastic behavior. A third-order low-pass Butterworth filter was applied to all *CF^D^* measurements to avoid confounding effects from load cell noise. This filter was able to minimize noise, while appropriately preserving the morphology of the force curve.

Furthermore, the percentage of the force relaxation of the distraction callus was estimated over time in each individual’s daily distraction force record. This parameter was calculated as the ratio of the daily (*day* = 1, 2, 3, … , 15) post-distraction force registered every 2 minutes (*min* = 2, 4, 6, … , 20) during the test (*CF* ^*D*^_*min*_^*day*^), to the daily distraction force peak (*CF* ^*D*^_*peak*_^*day*^) registered at the distraction performance (Equation 6).

$$R_{\min}^{day}=\left(1-\frac{{CF}_{\min}^{Dday}}{{CF}_{peak}^{Dday}}\right)\cdot 100\;\%$$ (6)

A mean individual force relaxation value (*R*_*min*_) was calculated every 2 minutes from the daily force relaxation (*R*_*min*_^*day*^) measured in each sheep during the distraction phase (Equation 7).

$$R_{\min}=\frac{\sum_{day=1}^{15}R_{\min}^{day}}{15}$$ (7)

### 2.4 Gait analysis

Each sheep was led through a gait corridor in which it stepped on the force platform with the operated limb at least 10 times per test during the consolidation phase (2–3 gait tests were conducted per week and animal). Footsteps performed at speeds outside the amble range (velocity <2 km/h or >4 km/s) or with interruptions were visually excluded (Mora-Macías et al., 2015a; Mora-Macías et al., 2015b; Blázquez-Carmona et al., 2021a; Blázquez-Carmona et al., 2023b). A mean value per test of the fixator force (*FF*, Equation 2) and *MGRF* was determined using the load cells and force platform, respectively. From these data, the distraction callus bearing capacity (*CF* ^*C*^, Equation 4) and stiffness (*K*_*C*_, Equation 5) were indirectly and quantitatively estimated in each test.

### 2.5 X-ray imaging

The mineralization and maturation of the osteoporotic distraction calluses were qualitatively assessed through periodic plantaromedial and dorsomedial x-ray imaging. It allowed monitoring the bone fragment displacement throughout the bone gap during the distraction phase and the callus mineral formation and bridging during the consolidation phase.

### 2.6 Viscoelastic model of the premineralized distraction callus

A viscoelastic model was used to evaluate the mechanical behavior of the premineralized distraction callus during the distraction phase. During this early-stage bone regeneration phase, the premineralized callus tissue is mainly composed of the organic matrix, divided into collagen fibers and ground substance.

The distraction force (*CF* ^*D*^) records were modeled using a generalized Maxwell model of a standard linear solid following the methodology outlined by Blázquez-Carmona et al. (2021b). This is a commonly used rheological model for numerous viscoelastic materials, including the premineralized distraction callus (Sopakayang and De Vita, 2011; Braunsmann et al., 2014; Blázquez-Carmona et al., 2021b). As illustrated in the spring-dashpot scheme in Figure 2D, the mechanical model of the premineralized distraction callus considers a linear elastic spring (stiffness *K*_*cf*_) representing the stationary response provided by the collagen fibers (*cf*). Meanwhile, the transient mechanical behavior of the early-stage callus, provided by the ground substance (*gs*) was modeled as a linear elastic spring (stiffness *K*_*gs*_) in series with a dashpot (damping constant *η*_*gs*_), both in parallel with *K*_*cf*_. Under the hypothesis that viscoelastic behavior is dominated by the organic matrix, this three-parameter model allows drawing significant conclusions about the apparent mechanical behavior of the premineralized distraction callus. Considering a constant distraction rate of 1 mm/day for the incremental bone fragment, the *CF* ^*D*^ can be expressed using the viscoelastic model parameters of the premineralized distraction callus, as defined in Equation 8.

$${CF}^{D}\left(t,T\right)=\left(K_{cf}\left(T\right)+K_{gs}\left(T\right)\cdot e^{-\frac{t}{\tau_{gs}\left(T\right)}}\right)\cdot u_{0}$$ (8)

were *t* is the relaxation time after the distraction performance, *T* is the distraction day, *τ*_*gs*_ is the relaxation time of the ground substance (*η*_*gs*_/*K*_*gs*_) and *u*_*0*_ is the callus elongation (1 mm/day). At the distraction performance (*t* = 0), the mathematical expression can be simplified as specified in Equation 9.

$${CF}^{D}\left(0,T\right)={CF}_{peak}^{D}\left(T\right)=\left(K_{cf}\left(T\right)+K_{gs}\left(T\right)\right)\cdot u_{0}$$ (9)

These viscoelastic model parameters were used to fit mathematically the daily monitorization of the *CF* ^*D*^ records. From each measurement, five points per 2 minutes (50 points in 20 min post-distraction) uniformly distributed were extracted and fitted to the solution of Equation 8, using a least-square algorithm. The curve fittings were performed in MATLAB R2023b^®^ (The MathWorks Inc., Natick, MA, USA) with ± 10% constraints on the peak force and the force at 20 min after the distraction performance.

### 2.7 Statistical analyses

The statistical analyses were performed using MATLAB R2023b^®^ (The MathWorks Inc., Natick, MA, USA). The data and correlations of the non-osteoporotic group were obtained from previous studies (Mora-Macías et al., 2015a; Mora-Macías et al., 2015b; Mora-Macías et al., 2016; Blázquez-Carmona et al., 2021b). Comparisons with these studies can be rigorous because they used the same bone animal model (sheep’s right hind metatarsus), distraction protocol (15 mm diaphyseal defect), experimental procedures, measurement techniques and animal conditions, but without considering osteoporosis induction.

For the distraction force record and viscoelastic model parameters, a mean and standard deviation value from the individual’s data were calculated each distraction day for osteoporotic and non-osteoporotic groups. The group’s mean and standard deviation data of the force relaxation (*R*) are obtained from a mean individual force relaxation value (*R* *_min_*) was calculated osteoporotic individuals. All underlying osteoporotic individual data are compiled in the Supplementary Material (Supplementary Tables S1-S17). The datasets of both groups were analyzed to check normal distribution using the Shapiro–Wilk test. This test showed that several datasets presented a non-normal distribution (*p* < 0.05). Consequently, the significance between groups was evaluated using the non-parametric test. Specifically, the non-parametric test Mann-Whitney U was selected as it aligns the distribution nature of both unpaired group’s data. The differences between groups were considered significant when *p* < 0.05. All *p-values* determined to evaluate the significance between groups are compiled in the Supplementary Material (Supplementary Tables S18-S21).

## 3 Results

### 3.1 Distraction force measurement

**FIGURE 3:**
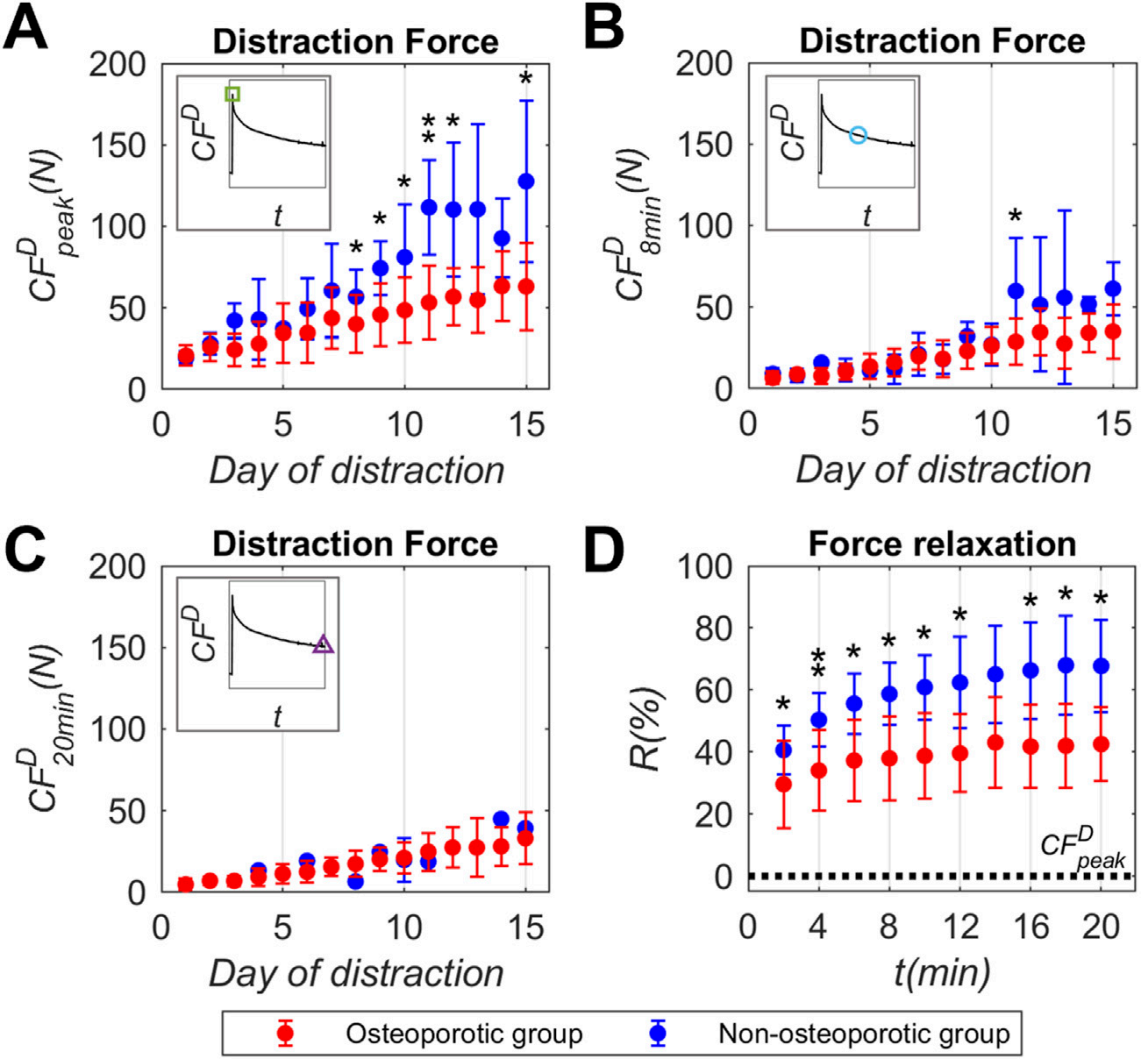
Callus distraction force (*CF* ^*D*^) record experimentally measured in osteoporotic (red) and non-osteoporotic (blue) sheep groups throughout the days of the distraction phase: **(A)** callus distraction force peak (*CF* ^*D*^_*peak*_), **(B)** callus distraction force at 8 min post-distraction (*CF* ^*D*^_*8min*_), **(C)** callus distraction force at 8 min post-distraction (*CF* ^*D*^_*20min*_), **(D)** mean individual percentage of distraction force relaxation (*R*) reported each 2 min of the relaxation time. Data presented as mean ± standard deviation values of the underlying individual osteoporotic sheep data, compiled in the Supplementary Material (Supplementary Tables S1-S4). Non-osteoporotic data obtained from Mora-Macías et al. (Mora-Macías et al., 2016). Significance evaluated by Mann-Whitney U test. * means *p* < 0.05 and ** means *p* < 0.01. Statistical analyses compiled in the Supplementary Material (Supplementary Tables S18-S19).

Figure 3A shows the mean and standard deviation values of distraction force (*CF* ^*D*^) in the osteoporotic group’s (red data) at the distraction performance (*CF* ^*D*^_*peak*_) over the days of the distraction phase. Results in a non-osteoporotic group (blue data (Mora-Macías et al., 2016)) are also included for further comparisons. For the same purpose, similar distraction force records at 8- and 20-min after the distraction performance (*CF* ^*D*^_*8min*_ and *CF* ^*D*^_*20min*_, respectively) were also represented in Figures 3B,C. The underlying distraction force datasets are compiled individually in the Supplementary Material (Supplementary Tables S1-S4).

Mean values of peak forces (*CF* ^*D*^_*peak*_, Figure 3A) reported in the osteoporotic group increased from 20.7 N to 63.1 N during the distraction phase. Regarding the record at 8 min post-distraction (*CF* ^*D*^_*8min*_, Figure 3B), the osteoporotic group presented force levels lower than those reported at the distraction performance. Once again, the osteoporotic mean values increased throughout the distraction phase, from 6.5 N to 35.1 N. As for the record at 20 min post-distraction (*CF* ^*D*^_*20min*_, Figure 3C), the osteoporotic mean force values (4.8 N to 33.1 N) did not show differences between minutes 8 and 20.

The mean and standard deviation of the force relaxation (*R*, Figure 3D) with respect to the *CF* ^*D*^_*peak*_ value are displayed every 2 min of the relaxation time. In the osteoporotic group, relaxation mainly occurs up to minute 2 (29.5%), increasing smoothly up to minute 14 (43%). No relaxation differences were found from minute 14 to minute 20 (42.5%). The standard deviation of the data remained relatively consistent over the relaxation time, averaging around 14% with respect to mean values.

### 3.2 Viscoelastic model parameters of the premineralized distraction callus

**FIGURE 4:**
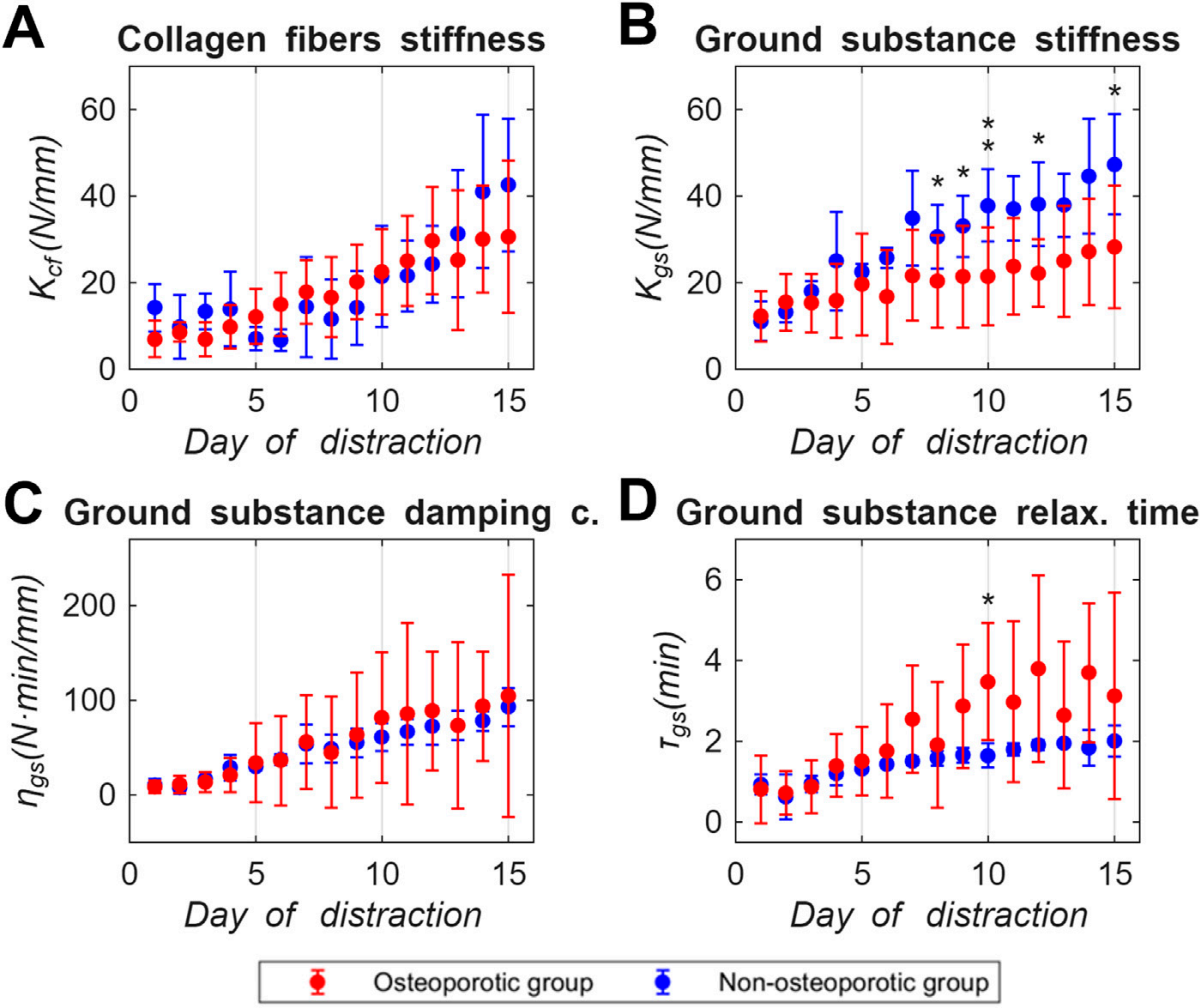
Viscoelastic model parameters of the premineralized distraction callus mechanical behavior in osteoporotic (red) and non-osteoporotic (blue) sheep groups throughout the days of the distraction phase: **(A)** collagen fibers stiffness (*K*_*cf*_), **(B)** ground substance stiffness (*K*_*gs*_), **(C)** ground substance damping constant (*η*_*gs*_), **(D)** ground substance relaxation time (*τ*_*gs*_). Data presented as mean ± standard deviation values of the underlying individual osteoporotic sheep data, compiled in the Supplementary Material (Supplementary Tables S5-S8). Non-osteoporotic data provided by Mora-Macías et al. (2016) and Blázquez-Carmona et al. (Blázquez-Carmona et al., 2021b). Significance evaluated by Mann-Whitney U test. * means *p* < 0.05 and ** means *p* < 0.01. Statistical analyses compiled in the Supplementary Material (Supplementary Table S20).

Figures 4A–D show the evolution of the collagen fibers stiffness (*K*_*cf*_) and ground substance stiffness (*K*_*gs*_), damping constant (*η*_*gs*_) and relaxation time (*τ*_*gs*_) of the osteoporotic group’s (red data) predicted by the viscoelastic model based on *CF* ^*D*^ measurements over the distraction phase. Non-osteoporotic data (blue data (Mora-Macías et al., 2016; Blázquez-Carmona et al., 2021b)) are also represented for further comparison reasons. Data is presented as the daily mean and standard deviation values between animals. The underlying viscoelastic parameters datasets are compiled individually in the Supplementary Material (Supplementary Tables S5-S8).

Regarding the collagen fibers stiffness (*K*_*cf*_, Figure 4A), the osteoporotic group exhibited an increase in stiffness from 7 N/mm to 30.6 N/mm during the distraction phase. Simultaneously, the standard deviation within the group increased throughout the days. For the ground substance, the stiffness (*K*_*gs*_, Figure 4B) was reported similar to the collagen fibers in the osteoporotic group, but showing a less significant increase from 12.2 N/mm to 28.3 N/mm during the distraction phase. The mean values of the ground substance damping constant (*η*_*gs*_, Figure 4C) increased throughout the distraction phase, reaching 104.8 N·min/mm at 15 days. The *η*_*gs*_ standard deviation with respect to mean values was higher than 1 in most of the days of the distraction phase (0.974 average). Regarding the ground substance relaxation time (*τ*_*gs*_, Figure 4D), the osteoporotic group also showed an increase in the mean values, reaching 2.9 min at 9 days of the distraction phase. From that point onward, the *τ*_*gs*_ mean and standard deviation remained relatively stable until the end of the distraction phase. The *η*_*gs*_ standard deviation was translated to the *τ*_*gs*_ standard deviation during the days of the distraction phase (0.655 average), due to the relationship between both parameters.

### 3.3 X-ray follow-up

**FIGURE 5:**
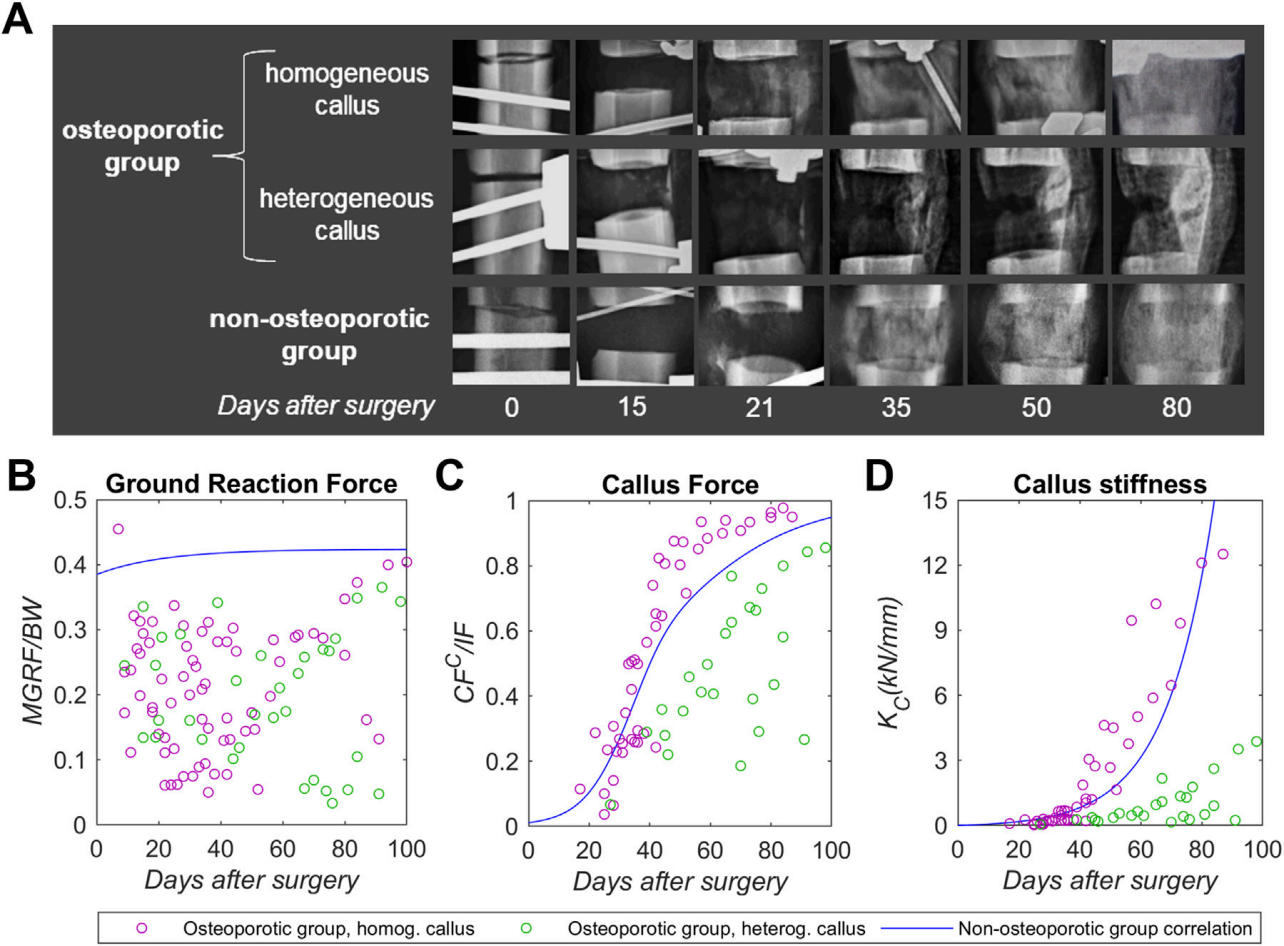
X-ray follow-up and gait mechanical parameters of the distraction callus in osteoporotic (with homogenous callus in purple, with heterogeneous callus in green), non-osteoporotic (blue correlation) sheep groups during the distraction osteogenesis experimental period: **(A)** plantaromedial x-ray imaging, **(B)** maximum ground reaction force with respect to the sheep body weight (*MGRF/BW*), **(C)** distraction callus bearing capacity (*CF* ^*C*^) normalized with the internal force (*IF*), **(D)** distraction callus longitudinal stiffness (*K*_*C*_). Underlying osteoporotic data compiled in the Supplementary Material (Supplementary Tables S9-S11). Non-osteoporotic x-ray imaging and correlations obtained from Mora-Macías et al. (Mora-Macías et al., 2015a; Mora-Macías et al., 2015b).

An x-ray follow-up of the osteoporotic group conducted at 0, 15, 21, 35, 50 and 80 days after the bone surgery is presented in Figure 5A. It shows representative examples of the distraction callus evolution: two from osteoporotic animals and one from non-osteoporotic (Mora-Macías et al., 2015b) animal as a non-pathologic reference. Qualitative analysis showed that none of the osteoporotic sheep reported non-union. 50% of the osteoporotic animals exhibited homogeneous or symmetrical callus mineralization, the remaining 50% displayed heterogeneous or asymmetrical callus formation. This osteoporotic subgroup with limited callus formation consistently showed localized bone formation primarily in the dorsolateral or the plantarolateral site of the limb.

### 3.4 Gait mechanical parameters of the callus

The results of the gait mechanical parameters (*MGRF*, *CF* ^*C*^ and *K*_*C*_) over the days after the bone surgery are presented in Figures 5B–D. Non-osteoporotic correlations (blue data (Mora-Macías et al., 2015a; Mora-Macías et al., 2015b)) are also depicted for comparative purposes. To elucidate gait dynamics differences between the osteoporotic group based on callus morphology, it was decided to split the gait analyses data into osteoporotic subgroup with homogeneous callus (purple data points) and osteoporotic subgroup with heterogeneous callus (green data points). The data of these parameters are compiled individually in the Supplementary Material (Supplementary Tables S9-S11).

The maximum ground reaction force (*MGRF*) data points were presented normalized with respect to the body weight (*BW*) to consider differences between individuals (*MGRF*/*BW*, Figure 5B). During the first 50 days after the bone surgery, the osteoporotic subgroups reported substantial data dispersion, ranging between 0.05 and 0.342. However, over the following 50 days, the *MGRF* of the osteoporotic subgroup with homogeneous callus seems to increase, while that of the osteoporotic subgroup with heterogeneous exhibited a declining trend. However, the dispersion of the data limited the identification of statistically significant differences. The callus bearing capacity (*CF* ^*C*^) progression was presented weighted to the internal force (*IF*) to consider differences between animals (*CF* ^*C*^/*IF*, Figure 5C). Both osteoporotic subgroups seem to report a recovery of the callus force over the days, although with different trends. The osteoporotic subgroup with homogeneous calluses presented an increase in *CF* ^*C*^, rising around 0.8 at 50 days and stabilizing around 0.96 at 80 days. In contrast, the osteoporotic subgroup with heterogeneous callus experienced an increase in *CF* ^*C*^ with an appreciably lower trend with wider dispersion, reaching around 0.35 at 50 days and 0.57 mean around 80 days. The callus stiffness (*K*_*C*_, Figure 5D) of the osteoporotic subgroups evolved with different trends. The osteoporotic subgroup with heterogenous callus showed an increasing trend of callus stiffening, ranging a mean value of 1.2 kN/mm at 80 days. Meanwhile, the osteoporotic subgroup with homogeneous calluses experienced an exponential trend in *K*_*C*_, reaching around 12.3 kN/mm at 80 days. Thus, the longitudinal stiffness of the heterogeneous osteoporotic callus represents 10 times less stiffness than that of the homogeneous osteoporotic callus at 80 days.

## 4 Discussion

The present study covers for the first time, an *in vivo* monitoring of the mechanical properties of the bone callus in osteoporotic subjects during its early phases of maturation. The osteoporotic ovine model and metatarsal DO protocol allows rigorous comparisons with the data obtained in previous similar studies in non-osteoporotic animal model and metatarsal DO protocol (Mora-Macías et al., 2015a; Mora-Macías et al., 2015b; Mora-Macías et al., 2016; Blázquez-Carmona et al., 2021b). Thus, the influence of osteoporosis on the ossification of DO-regenerated callus can be isolated and discussed.

Beginning with the mechanical comparison of the early premineralized distraction callus, its viscoelastic response to 1 mm/day traction for 15 days has been assessed by means of distraction force tests. The mean values of callus distraction force peak reported in the osteoporotic group (*CF* ^*D*^_*peak*_, Figure 3A) increased from 20.7 N to 63.1 N during the distraction phase. Meanwhile, the mean peak force values of the non-osteoporotic group (Mora-Macías et al., 2016) seem to double throughout the days of the same phase (from 19.5 N to 127.6 N). In this non-pathologic group, the variability of data increased over the days, and was higher than in the osteoporotic group. Significant differences between groups were found on most days of the second half of the distraction phase (*p* < 0.05 at days 8, 9, 10 and 15, and *p* < 0.01 at day 11). These force differences between groups over the distraction phase for the same elongations (1 mm of distraction per day) of the non-mineralized distracted callus show an impaired 50% mechanical capabilities due to the pathology. With respect to the force at 8 min post-distraction (*CF* ^*D*^_*8min*_, Figure 3B), both groups presented force levels considerably lower than those reported at the distraction performance, but the statistical differences became less significant. Once again, the osteoporotic and non-osteoporotic mean values increased throughout the distraction phase (osteoporotic from 6.5 N to 35.1 N, and non-osteoporotic from 9.1 N to 61.3 N). Although *CF* ^*D*^_*8min*_ standard deviation was generally lower than that of *CF* ^*D*^_*peak*_ recording in both groups, significant differences were only found at day 11 (*p* < 0.05). As for the force at 20 min post-distraction (*CF* ^*D*^_*20min*_, Figure 3C), the osteoporotic mean force values (from 4.8 N to 33.1 N throughout the distraction phase) did not show differences with respect to minute 8. In contrast, distraction forces from non-osteoporotic animals continued to decrease to osteoporotic-range values during those 12 min. In this sense, no significant force differences were found between both groups post 20 min from the distraction performance. Force relaxation (*R*, Figure 3D) normalized to the *CF* ^*D*^_*peak*_, occurred primarly during the first 2 min after distraction in both groups, but to a lesser extent in the pathological group (29.5%) compared to the non-osteoporotic group (40.6%). In the osteoporotic group, the force relaxation progresses smoothly, reaching a near-stable state by minute 14 (43% average relative to the *CF* ^*D*^_*peak*_). In contrast, the non-osteoporotic group exhibit a more pronounced relaxation stabilizing around minute 14 (65.0%). Significant differences between groups were found across most relaxation time points (*p* < 0.05), with an average group difference of over 18% from minute 6 onwards. According to Blázquez-Carmona et al. (Blázquez-Carmona et al., 2021b), the callus distraction force remains constant from minute 20. Thus, the reported force level tends to be mainly due to the elastic component of the premineralized distraction callus from that time. In addition, there is quick and limited relaxation of the osteoporotic group compared to non-pathological animals. Both insights seem to agree that osteoporosis could alter the viscoelastic components of the premineralized osteoporotic callus. This fact appears to be responsible for the differences found in permineralized callus viscoelastic competences (*CF* ^*D*^_*peak*_, Figure 3A) with respect to the non-osteoporotic group previously mentioned.

The aforementioned differences can be further explored through the viscoelastic model parameters of the premineralized distraction callus, providing insights into the mechanical behavior of its fundamental organic matrix compounds: the collagen fibers and the ground substance, both synthetized by osteoblasts. The stiffness of the collagen fibers (*K*_*cf*_, Figure 4A) enhanced from average values of 7 N/mm to 30.6 N/mm throughout the distraction phase. Meanwhile, the collagen fibers of the non-osteoporotic group (Mora-Macías et al., 2016; Blázquez-Carmona et al., 2021b) experienced an almost similar average stiffening, from 14.2 N/mm to 42.6 N/mm. Although no significant differences were found between groups, the osteoporotic group exhibited lower average *K*_*cf*_ values during the final days of the distraction phase. A possible explanation for these differences is an osteoporosis-induced reduction in osteoblastic collagen synthesis (Zhang et al., 2018). On the other hand, the groups showed significant differences in ground substance stiffening (*K*_*gs*_, Figure 4B) with similar standard deviation (*p* < 0.05 at 8, 9, 12 and 15 days, and *p* < 0.01 at 10 days). Specifically, the osteoporotic group experienced an average *K*_*gs*_ increase with a lower slope than that reported by the non-osteoporotic group, from 12.2 N/mm to 28.3 N/mm and from 11.1 N/mm to 47.4 N/mm, respectively. The results reveal that the elastic component of the ground substance seems to be weakened by osteoporosis. This can be supported by the significant differences found in callus distraction force peak (*CF* ^*D*^_*peak*_, Figure 3A). According to the mathematical expression presented in Equation 9, for virtually similar levels of collagen fiber stiffening (*K*_*cf*_) and same callus elongations (*u*_*0*_ = 1 mm), the reason for diminished osteoporotic *CF* ^*D*^_*peak*_ must be lower ground substance stiffness values (*K*_*gs*_). This finding may also be a consequence of impaired osteoblastic function, decreasing the synthesis of proteoglycans and glycosaminoglycans contained in the ground substance (Tong et al., 2013). With respect to the viscous behavior of the ground substance (*η*_*gs*_, Figure 4C), the osteoporotic group and the non-osteoporotic group presented a similar average increase, from 8.7 to 104.8 N·min/mm and from 10.6 to 92.9 N·min/mm, respectively. The difference between the groups can be appreciated in the standard deviation values. Whereas the osteoporotic group showed an increase in standard deviation, from 6.4 N·min/mm to 127.8 N·min/mm, the non-osteoporotic group presents low and constant values of this statistical parameter, 12.2 N·min/mm average, compared to the osteoporotic group. The standard deviation level of both groups with respect to the mean values was also transferred to the relaxation time of the ground substance (*τ*_*gs*_, Figure 4D), since these parameters are linearly correlated with each other. The non-osteoporotic group increased its mean relaxation time from 0.92 to 2.00 min throughout the distraction phase. In contrast, the increase in mean *τ*_*gs*_ of the osteoporotic group has a higher tendency, from 0.81 to 3.13 min. The differences between groups (*p* < 0.05 at 10 days) are mainly related to an impaired elastic component of the osteoporotic ground substance (*K*_*gs*_, Figure 4B) as a hypothetical consequence of an alteration in proteoglycans and glycosaminoglycans synthesis due to the disease (Tong et al., 2013). Glycosaminoglycans are also responsible for regulating water content, giving the organic matrix its damping competence. According to the variability reported in the damping constant (*η*_*gs*_, Figure 4C), it seems that the alteration of this substance varies among pathological individuals.

The distraction callus mineralization of the osteoporotic group was qualitatively assessed through periodic x-ray imaging (Figure 5A) and compared with the non-osteoporotic group (Mora-Macías et al., 2015b). The osteoporotic subgroup with homogeneous callus seems to present similar mineral formation to the non-osteoporotic group from the end of the distraction phase (at 21 days after surgery, Figure 5A). On the other hand, the osteoporotic subgroup with heterogeneous callus presents limited mineralization in the lateral site of the bone, close to its main dorsal or plantar blood vessels. Thus, a lack of mineral formation was observed in the medial site of the bone in these pathologic animals. These intra-group radiographic differences have not been observed in previous DO studies conducted in small osteoporotic animal models (Arslan et al., 2003; Tatehara et al., 2011; Sun et al., 2014), which evidences the discrepancies with large osteoporotic animal models.

**FIGURE 6:**
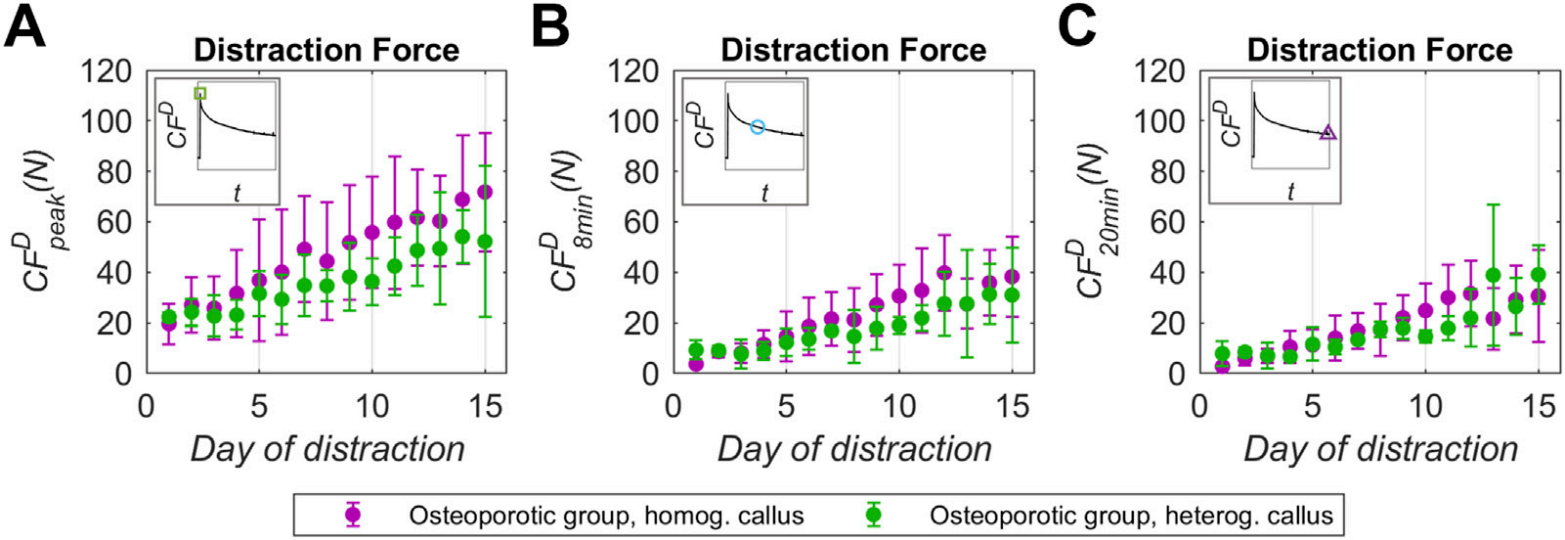
Distraction force (*CF* ^*D*^) record experimentally measured in osteoporotic sheep group with homogeneous callus (purple) and heterogeneous callus (green) throughout the days of the distraction phase: **(A)** distraction force peak (*CF^D^_peak_*), **(B)** force relaxation at 8 min post-distraction (*CF^D^_8min_*), **(C)** force relaxation at 20 min post-distraction (*CF^D^_20min_*). Data presented as mean ± standard deviation values of the underlying individual osteoporotic sheep data, compiled in the Supplementary Material (Supplementary Tables S12-S17). Significance evaluated by Mann-Whitney U test. * means *p* < 0.05 and ** means *p* < 0.01. Statistical analyses compiled in the Supplementary Material (Supplementary Table S21).

The gait mechanical parameters (*MGRF*, *CF* ^*C*^, and *K*_*C*_, Figures 5B–D) quantitatively supported the x-ray findings (Figure 5A). The maximum ground reaction force with respect to the body weight (*MGRF*/*BW*, Figure 5B) reported by the osteoporotic subgroups, are half those of the non-osteoporotic group during the first 50 days after surgery (data dispersion around 0.2 mean of the osteoporotic subgroups compared with 0.4 of the non-osteoporotic correlation). It appears that the induced osteoporosis differently affects the animals' confidence to perform footsteps. During the remaining 50 days, the osteoporotic subgroup with heterogeneous callus continues with similar mean and dispersion levels. In contrast, the homogeneous callus subgroup appears to have a *MGRF* recovery tendency towards the non-osteoporotic correlation. As for the callus force weighted to the internal force (*CF* ^*C*^/*IF*, Figure 5C), the osteoporotic subgroup with homogeneous callus showed a recovery of callus bearing capacity similar to the non-osteoporotic group. Compared to both groups, the osteoporotic subgroup with heterogeneous callus presented a slower and more variable functional recovery. In terms of mineralization level, the callus compression stiffness (*K*_*C*_, Figure 5D) also showed different trends between the osteoporotic subgroups. From early stages, the osteoporotic group with homogeneous callus reported an exponential trend that appears to be aligned with the non-osteoporotic group correlation. On the other hand, the osteoporotic group with heterogeneous callus seems to present an impaired lineal stiffening trend in comparison with previously mentioned groups (10-fold less stiffness at 80 days). The findings seem to correlate with the x-ray follow-up results (Figure 5A) in showing a mineral progression of the osteoporotic subgroup with homogenous callus similar to the non-osteoporotic group. This may indicate that the fixator could be removed in this osteoporotic subgroup at equivalent days after surgery to those of the non-osteoporotic group. In contrast, the woven bone mineralization and osteons formation appear to be severely compromised in the osteoporotic subgroup with heterogenous callus, thus requiring prolonged external fixation. This impaired level of mineralization and callus bearing capacity in 50% of the osteoporotic subjects may be related to the early stages of the premineralized callus. Figures 6A–C shows the distraction force peak and force relaxation responses at 8 and 20 min between both osteoporotic subgroups throughout the distraction phase. The osteoporotic subgroup with heterogeneous callus presented lower mean levels of peak force and force relaxation at 8 min (although without significant differences), finally equalized at 20 min. Based on these callus distraction force differences, the osteoporotic group with heterogeneous callus may exhibit a further delay in organic matrix synthesis that would subsequently be reflected in the later mineral apposition rate (Young, 2003; Zhang et al., 2018; Rupp et al., 2021).

This study has some limitations that should be mentioned. Regarding the number of animals, five of them suffered health complications during the osteoporosis induction phase or following the bone surgery, which forced them to be prematurely sacrificed. These individuals did not report data, and they were not considered when performing the statistical analysis. The sample size of large animals is limited due to their high complexity and economic cost (e.g., in surgeries and management), as well as the extended study periods required. However, their strong translational relevance to humans makes each reported data highly valuable. With respect to the distraction force test, 15% of force measures had to be rejected or stopped due to technical problems with the data acquisition system or load cells, as well as uncontrolled animal movements. The tests did not last more than 20 min from the distraction performance to ensure animal welfare. For the same reason, the number of gait tests per animal was limited to 2 or 3 per week. In addition, three sheep were excluded from this assessment as they were unable to perform stance phases at speeds within the amble range or without interruptions.

In conclusion, this work reports novel translational quantitative results on the influence of osteoporosis on the mechanical properties of the distraction callus under bone regeneration using the metatarsal DO ovine model. *In vivo* monitoring of the instrumented fixator in combination with x-ray follow-up allows a detailed study of the evolution of the callus ossification process from its earliest stages. During the distraction phase, osteoporosis was found to reduce the viscoelastic competences of the premineralized callus by up to 50%. According to the viscoelastic model of DO, this reduction appears to stem from a weakened elastic component of the ground substance. This seems to be related to a reduction of the osteoblastic function due to osteoporosis, limiting and altering the proteoglycans and glycosaminoglycans synthesis. As for the consolidation phase, the x-ray imaging showed that osteoporosis could induce a limited and focused mineralization in more vascularized sites. The gait analyses align with the x-ray follow-up, revealing notably impaired mechanical parameters of the woven tissue compared to non-osteoporotic individuals. However, some osteoporotic patients may present a similar ossification pattern to non-osteoporotic individuals, allowing the fixator removal at a similar time point. These differences in woven bone mineralization among osteoporotic patients appear to be associated with the early premineralized stages, exhibiting altered callus viscoelastic properties probably due to a further delay in organic matrix synthesis. In this regard, further research is necessary to rigorously understand the mechanobiological causes of these differences among osteoporotic individuals. However, these highly translational data allow to extend the knowledge on osteoporotic bone regeneration, especially for clinical cases of secondary osteoporosis. In clinical feasibility terms and according to the findings of the present work, patients would have a high probability of consolidation delay and heterogenous mineralization for a critical-size bone defect. In addition, the information provided will help in the development of numerical models to *in silico* characterize and predict the progression of the mechanical behavior of the DO woven tissue in the presence of osteoporosis.

## Data Availability

The original contributions presented in the study are included in the article/Supplementary Material, further inquiries can be directed to the corresponding author.
