# Supplementary material for: Bone regeneration during osteoporosis: a translational in vivo monitoring of callus mechanical parameters
Source: Front Bioeng Biotechnol. 2025 Oct 29;13:1646500. doi: 10.3389/fbioe.2025.1646500 (PMC12605322; doi:10.3389/fbioe.2025.1646500)
Supplement: Supplementary file 1 [file DataSheet1.docx]

**Supplementary Material Index**

[Supplementary Material 1. Data Acquisition System 2](#_Toc210389903)

[Supplementary Table S1. Underlying osteoporotic sheep data points of Figure 3A. 12](#_Toc210389904)

[Supplementary Table S2. Underlying osteoporotic sheep data points of Figure 3B. 13](#_Toc210389905)

[Supplementary Table S3. Underlying osteoporotic sheep data points of Figure 3C. 14](#_Toc210389906)

[Supplementary Table S4. Underlying osteoporotic sheep data points of Figure 3D. 15](#_Toc210389907)

[Supplementary Table S5. Underlying osteoporotic sheep data points of Figure 4A. 16](#_Toc210389908)

[Supplementary Table S6. Underlying osteoporotic sheep data points of Figure 4B. 17](#_Toc210389909)

[Supplementary Table S7. Underlying osteoporotic sheep data points of Figure 4C. 18](#_Toc210389910)

[Supplementary Table S8. Underlying osteoporotic sheep data points of Figure 4D. 19](#_Toc210389911)

[Supplementary Table S9. Osteoporotic sheep data points of Figure 5B. 20](#_Toc210389912)

[Supplementary Table S10. Osteoporotic sheep data points of Figure 5C. 21](#_Toc210389913)

[Supplementary Table S11. Osteoporotic sheep data points of Figure 5D. 22](#_Toc210389914)

[Supplementary Table S12. Underlying osteoporotic sheep with heterogeneous callus data points of Figure 6A. 23](#_Toc210389915)

[Supplementary Table S13. Underlying osteoporotic sheep with homogeneous callus data points of Figure 6A. 24](#_Toc210389916)

[Supplementary Table S14. Underlying osteoporotic sheep with heterogenous callus data points of Figure 6B. 25](#_Toc210389917)

[Supplementary Table S15. Underlying osteoporotic sheep with homogenous callus data points of Figure 6B. 26](#_Toc210389918)

[Supplementary Table S16. Underlying osteoporotic sheep with heterogenous callus data points of Figure 6C. 27](#_Toc210389919)

[Supplementary Table S17. Underlying osteoporotic sheep with homogenous callus data points of Figure 6C. 28](#_Toc210389920)

[Supplementary Table S18. Statistical analysis of Figure 3A-C. 29](#_Toc210389921)

[Supplementary Table S19. Statistical analysis of Figure 3D. 30](#_Toc210389922)

[Supplementary Table S20. Statistical analysis of Figure 4A-D. 31](#_Toc210389923)

[Supplementary Table S21. Statistical analysis of Figure 6A-C. 32](#_Toc210389924)

# Supplementary Material 1. Data Acquisition System

***Data acquisition system overview***

An advanced monitoring system has been designed to collect data from the load cells attached to the bone-fixator system. Traditionally, wired communications have been used to transmit information because they offer a good level of reliability and security. Nevertheless, this approach has constraints since the electronic board is located above the sheep and excessive wiring makes the system difficult to operate. Thus, a wireless connectivity via Wi-Fi has been implemented to transmit the data remotely, and the device has been equipped with all the components necessary to provide sensing, computation, real-time monitoring, communication for data collection, processing, transmission, storage, visualization and analysis. Moreover, the data acquisition system fulfils the main concerns of IoT (Internet of Things) devices, as it is compact, cost-effective, battery-powered, microcontroller-based, and has sufficient autonomy. It is easy to handle and prevents data loss during the animal experimental procedures. The electronic board begins collecting data through commands it receives from a remote PC running the control software.

***Hardware description***

Figure SM1 shows the general block diagram of the hardware used for the developed device. The system basically consists of conditioning interfaces of six load cells, a data processing module (microcontroller) that collects all the measurement parameters, and an energy module. The data recorded from the force sensors are received and processed by a microcontroller, and subsequently transferred to a PC using a wireless transceiver via Wi-Fi. The PC was used for data handling and access to a monitoring application.


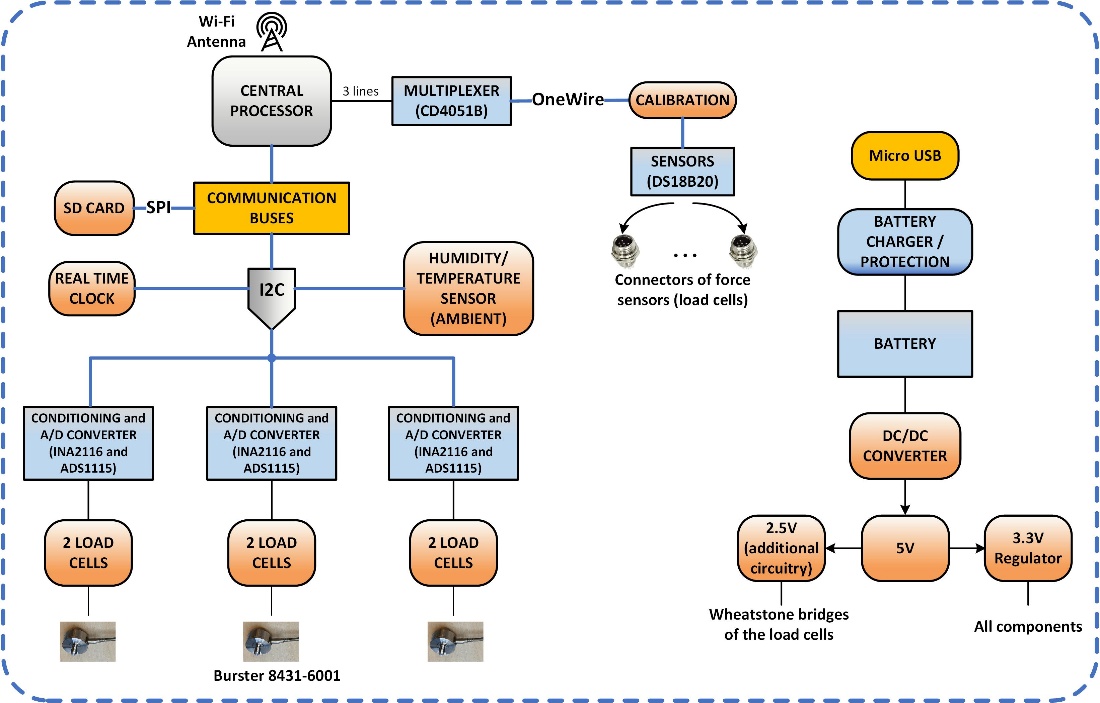


**Figure SM1** Block diagram of the architecture of the electronic board.

*Unit control.* The main component of the data acquisition system is a 32-bit ESP32 processor from Espressif Systems. This system-on-chip, leverages a dual-core processor and Real-Time Operating System (RTOS) to enable the simultaneous execution of multiple tasks. It also features peripheral interfaces such as SPI and I2C for connecting various external components. Additionally, it offers wireless connectivity options, including Bluetooth and a Wi-Fi transceiver, enabling remote data communication.

*Conditioning interfaces*. For the electronics acquisition and conditioning, external A/D converters are included instead of the ESP32's internal converters. Although the ESP32's internal converter is faster, it does not provide sufficient precision, as it only has a 10-bit resolution. Thus, three ADS1115 16-bit A/D converters, connected to the microcontroller via a standard I2C bus, have been chosen. These converters are suitable for measuring four single-ended inputs with 15-bit resolution or two differential inputs with 16-bit resolution, with a maximum sampling rate of 250 samples per second. INA2116® (Texas Instruments, Texas, EEUU) precision differential instrumentation amplifiers are used to adapt the signals from the six load cells to the A/D converters. The differential scheme provides greater noise rejection.

The ADS1115 A/D converter includes a channel selector with a relatively long settling time; therefore, when switching channels, it is necessary to wait for the signal to stabilize before initiating digitization. By using multiple units operating in alternation, the available time for the converter's input switch can be extended, ensuring signal stability. A separate ground plane has been designed to improve system interference rejection and in order to place the filtering components close to the active components. In this configuration, all the A/D converter grounds are isolated from the main ground plane of the board and are connected to it at a single point only.

On the other hand, additional circuitry has been used. Since force sensors operate at 2.5 V, a linear regulator with a pass transistor was designed to generate this voltage from a precision voltage reference, ensuring a stable and accurate supply. The circuit incorporates short-circuit protection on the connecting wires. Moreover, a real-time clock (DS3231) has also been connected to the I2C bus to maintain the system’s time and date. An SD card allows the collected data to be stored locally, which can then be accessed remotely through SPI bus using the PC's control software. A humidity and temperature sensor (SHT11) has been included to monitor environmental conditions.

Finally, the user-friendly operation of the six load cells has been simplified by making the force probes interchangeable across all six connectors. This requirement was introduced to enable the detection of each load cell and to perform calibration that considers the variations between different force sensors of the same type. To achieve this, a DS18B20 temperature sensor has been placed inside each GX12 connector. However, the sensor was not used to measure temperature, but rather to leverage the unique serial number provided by the manufacturer, allowing each force sensor to be individually identified. This is a method that simplifies the solution compared to more complex circuitry. Only one of the six pins on the GX12 connector is needed for operation, as the DS18B20 uses the OneWire bus from Maxim. A CD4051B multiplexer, controlled by the ESP32 microcontroller, switches the readings from each of the six temperature sensors. Therefore, once a force sensor has been calibrated using the Windows user application, this calibration data can be stored along with the sensor's code. When the program detects the sensor code, it searches the calibration database and automatically applies the appropriate calibration tables.

*Power supply system*. The energy module allows the entire system to operate on battery power, thereby avoiding the need for a connection to the electrical grid. It consists of a Lithium battery type 18650, a battery charging circuit, a protection circuit, and voltage regulation. The data acquisition system operates at three different voltages: 2.5 V, 3.3 V and 5 V. The interfaces for the signal conditioning and the circuit that provides the reference voltage of 2.5 V for the six Wheatstone bridges of the load cells require 5 V, whereas the microcontroller operates at 3.3 V. Since the battery voltage varies between 2.9 V and 4.2 V, a DC-DC boost circuit (SDB628) was used to convert the varying battery voltage into a stable 5 V output. From this, a AS1117-3.3 LDO regulator is used to power the microcontroller. The SDB628 converter can deliver up to 1 A of current. When Wi-Fi is enabled, the peak current demand reaches approximately 400 mA, which can be readily supplied by the LDO regulator connected to the boost converter output. Finally, the TP4056 IC is used to charge the battery and keep it within its operating limits. An additional circuit with the FS8205A and FS312F-G ICs is used for battery protection, consisting of a voltage detector and a feed-through MOSFET.

Low-cost and low-power commercial off-the-shelf components were employed in the hardware design to ensure availability, provide sufficient accuracy for extracting relevant experimental data, and guarantee adequate autonomy during each animal test.

Two electronics printed circuit boards have been manufactured: a main board of dimensions 10 cm (L) x 7 cm (W), which includes all the necessary functionality, and a secondary board designed to facilitate the distribution and wiring of six connectors to the main board, accommodating the extensive number of interconnections required. Six-pin GX12 connectors have been included; they are robust, metallic, economical, waterproof, and allow the load cells to be threaded, preventing disconnection caused by animal movements, thereby enhancing the reliability of data recording. Figure SM2A illustrates the electronic 3D design, while the details of the hardware implementation are shown in Figure SM2B. The device is encapsulated in a waterproof metallic enclosure for high robustness. The dimensions of the complete assembly are 19 cm (L) × 12 cm (W) × 4 cm (D).


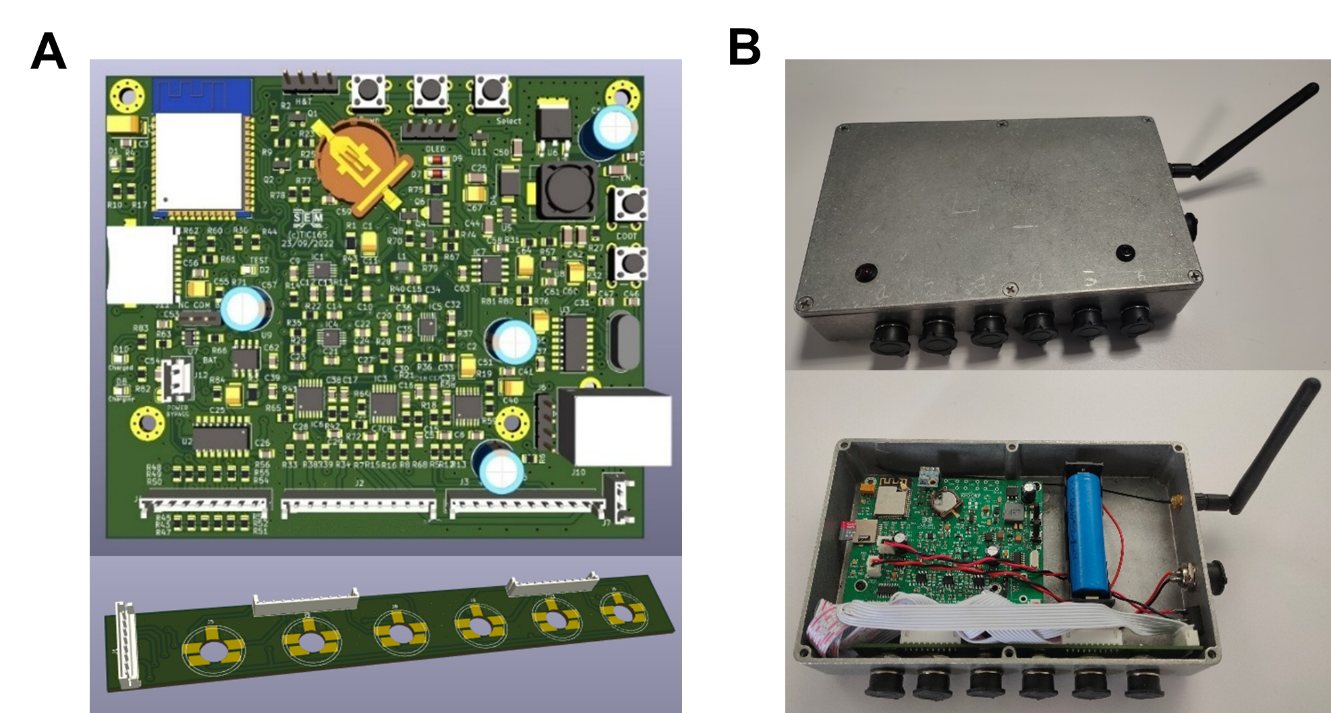


**Figure SM2** Hardware design. (**A**) 3D view of the two PCBs. (**B**) Assembly and packaging.

***Microcontroller firmware***

The firmware is responsible for managing the components on the electronic board and establishing communication with the user's control software via Wi-Fi. Upon powering on the system, the firmware initializes the electronic devices and the microSD card, and subsequently flashes an LED to indicate that the system is running. It then checks for a registered Wi-Fi network nearby; if one is found, it connects as a client, updates the clock in real time, and waits for commands to start operating. If no Wi-Fi network is found, the device creates its own network to which the user can connect to use the PC control software application. The ESP32 microcontroller used integrates two processors with shared memory. The firmware was programmed using the FreeRTOS real-time operating system. This system has a task scheduler that allows multiple tasks to be executed simultaneously and automatically distributed between the two processors.

As mentioned above, in order to detect the force sensor and perform a calibration that considers the variations between different force sensors of the same type, DS18B20 temperature sensors are placed in each GX12 connector. These temperature sensors provide a unique serial number that identifies each force sensor. Only one pin of the sensor connector is required for operation, as it uses the MAXIM manufacturer's ONEWIRE bus. This way, each sensor is connected and identified before data acquisition, and the corresponding calibration can be applied after data collection in the control software. In addition to the calibration of each force sensor, an electrical calibration is also performed using linear interpolation for each of the six channels. This takes into account small variations in response from one channel to another so that the measurement result does not vary depending on the channel used for the load cells. This electrical calibration is applied directly to the electronic board (microcontroller) and is independent of the force calibration performed on a hydraulic testing machine. Additionally, the electrical calibration does not depend on the calibration of each load cell performed within the control software. Ultimately, the calibration performed in the microcontroller firmware ensures that all channels of the data acquisition system will behave identically from an electrical standpoint.

The microcontroller firmware configures several TCP/IP servers to interact with the system. First, there is an HTTP server for updating the system firmware. Second, there is a TELNET server for interactive access to the system through a local command interpreter. This allows for electrical calibration, sending commands, and monitoring system operation. Finally, there is a TCP server responsible for transferring the gathered data to the control software.

Regarding data gathering, the electronic board includes three A/D converter chips: ADC1, ADC2, and ADC3. Each chip manages two inputs, for a total of six channels, but each chip contains only one ADC. Thus, each chip must switch between two differential inputs that physically connect two wires. This differential input configuration reduces noise and provides a wider measurement range. Therefore, the A/D converters are used in multiplexed mode, allowing the operation of the three A/D converters to be interleaved, overlapping conversion times and achieving higher conversion speeds as shown in Figure SM3. The figure shows that, with a cadence of 14 ms between readings, approximately 72 samples per second can be obtained by multiplexing the three converters. This number of samples is sufficient for this application.


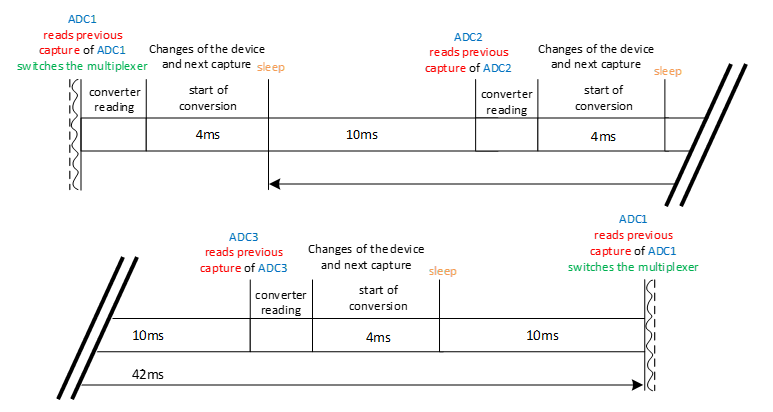
**Figure SM3** Sampling schedule for the three A/D converters.

The ESP32 microcontroller is sufficiently powerful for the target application, enabling it to run the program without overloading. CPU consumption measurements during the acquisition process under load have been performed, yielding values of around 20%.

***Control software***

The part of the data acquisition system that facilitates user interaction is the control software. This is a Windows-based program that runs on a PC. The program allows the user to perform the following actions: 1) start and stop data acquisition and calibrate each force sensor; 2) save the captured data and retrieve data from previous tests in CSV format; and 3) view the sampling progress in real time, with the ability to zoom in on a specific channel.

The program was developed using the Python language due to its speed and simplicity in managing data, graphics, and files. The Qt library was used to create the user interface, along with PyQtGraph for graphical application programming. The NumPy library was employed for mathematical analysis. Figure SM4 illustrates the developed monitoring application.


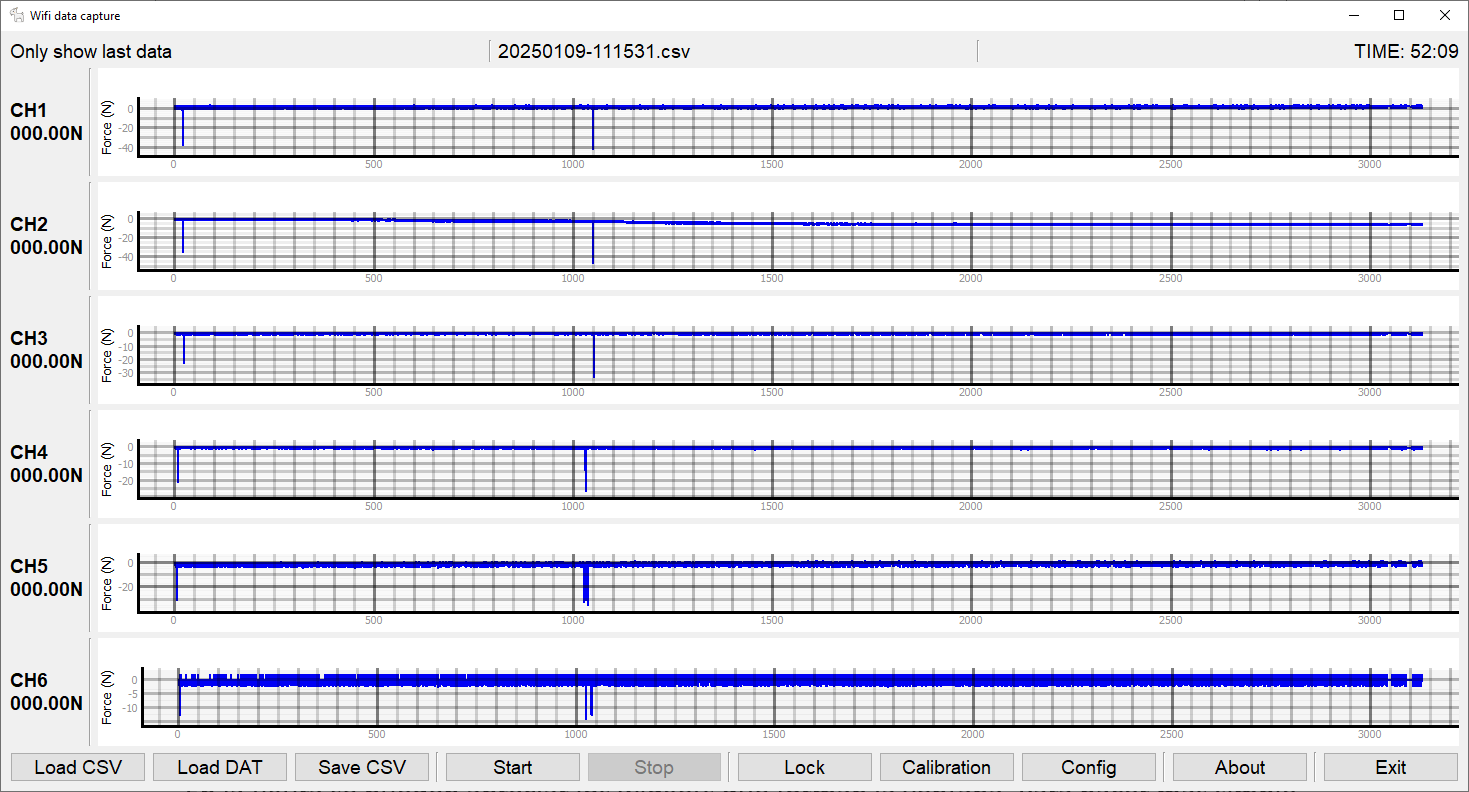


**Figure SM4** Graphical user interface for the monitoring application.

The program's primary purpose is to provide a real-time graphical display of data from force sensors placed remotely on the bone-fixator system of the sheep, functioning as a telemetry application. The process begins by pressing the "Start" button, which initiates a connection attempt with the remote electronic device. If the server's acknowledgment message is not received, the user is notified of the error, and the operation is canceled. Data collection is performed in three phases: 1) A connection is established with the remote device by configuring its IP address and using the TCP protocol to communicate with the server, which interprets the commands. 2) Data capture and transmission from the sensors are initiated; this data is interpreted using the interpolation functions corresponding to each sensor, and the data is stored in memory. 3) The received data is displayed graphically, allowing for immediate visualization that gives the user the impression of seeing the values in real time.

If a transmission error is detected or the stop button is pressed, data reception is interrupted. However, the graphs remain active so the user can analyze them, expand a channel, or save the information. The program allows force sensors to be calibrated in the laboratory using evenly spaced points corresponding to different loads applied to the sensors. Figure SM5 shows the configuration that enables a piecewise linear approximation between the A/D converter readings and the force values. The slope of this relationship depends on several factors, such as sensor type, mechanical structure, and temperature, making laboratory calibration necessary. The obtained calibration values are saved in a file on disk, which can be updated if the sensor configuration changes. These values can also be modified and recalibrated directly from the control software. As detailed, all parts of the data acquisition system were involved in the calibration process.


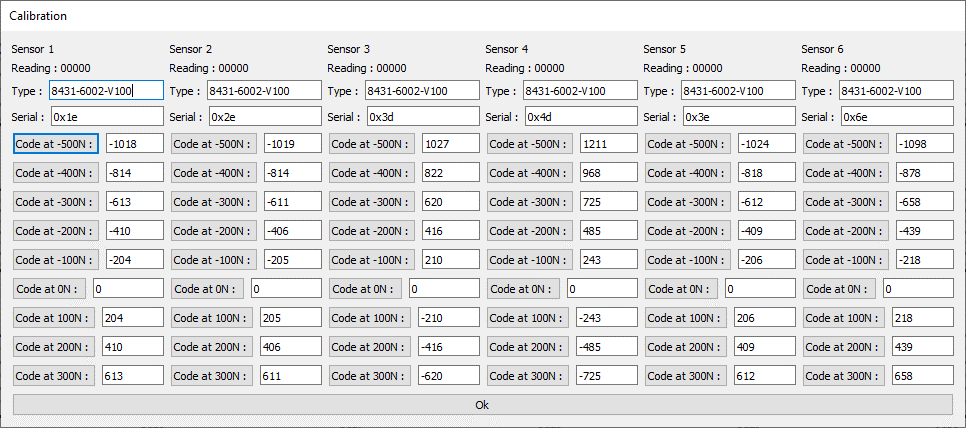


**Figure SM5** Graphical user interface for the calibration of the force sensors.

# Supplementary Table S1. Underlying osteoporotic sheep data points of Figure 3A.

| ***CF^D^_peak_*(N)** | **Distraction day** | | | | | | | | | | | | | | |
| --- | --- | --- | --- | --- | --- | --- | --- | --- | --- | --- | --- | --- | --- | --- | --- |
| **Sheep code** | **1** | **2** | **3** | **4** | **5** | **6** | **7** | **8** | **9** | **10** | **11** | **12** | **13** | **14** | **15** |
| 1 | - | - | 18.55 | 20.43 | 31.56 | 17.63 | - | 29.32 | 32.89 | - | - | - | 18.22 | - | 15.10 |
| 2 | - | 25.90 | 21.73 | - | - | 37.46 | - | - | - | - | - | - | 51.26 | - | 67.54 |
| 3 | 23.19 | 29.61 | 34.64 | 27.38 | 42.87 | 36.77 | 48.75 | 42.98 | 58.28 | 46.71 | 55.71 | 48.84 | 67.61 | 58.12 | 83.63 |
| 4 | 23.68 | 17.13 | 12.93 | 16.22 | 20.74 | 20.08 | 27.46 | 31.03 | 28.50 | 33.56 | 34.93 | 34.62 | 37.53 | 42.39 | 42.66 |
| 5 | 20.23 | 24.69 | 25.70 | 28.63 | 31.14 | 34.64 | 28.37 | 35.32 | 33.38 | 28.89 | 36.87 | 62.63 | 72.47 | 61.99 | - |
| 6 | 22.90 | 31.02 | 39.29 | 35.90 | 41.77 | 38.32 | 58.27 | 55.73 | 53.35 | 53.76 | 69.98 | 69.01 | 77.05 | 88.19 | 85.97 |
| 7 | 23.53 | 23.98 | 32.80 | 32.94 | 26.17 | 36.91 | 40.12 | 32.10 | 42.09 | 55.54 | 45.28 | 73.28 | 75.34 | 90.99 | 94.85 |
| 8 | 28.31 | 44.28 | 31.66 | 57.19 | 76.00 | 81.45 | 81.27 | 79.39 | 90.61 | 92.34 | 101.11 | 82.72 | 66.82 | 82.59 | 83.77 |
| 9 | 15.84 | 19.31 | 16.04 | 21.11 | 27.64 | 28.49 | 37.05 | 34.77 | 36.54 | 35.83 | 46.36 | 41.58 | 43.89 | 43.34 | 55.12 |
| 10 | 7.81 | 17.26 | 9.36 | 11.22 | 12.72 | 15.33 | 28.97 | 20.05 | 36.40 | 41.51 | 36.24 | 41.96 | 38.49 | 39.10 | 39.47 |
| Mean | 20.69 | 25.91 | 24.27 | 27.89 | 34.51 | 34.71 | 43.78 | 40.08 | 45.78 | 48.52 | 53.31 | 56.83 | 54.87 | 63.34 | 63.12 |
| Std | 6.28 | 8.50 | 10.11 | 13.53 | 18.18 | 18.66 | 18.60 | 17.72 | 19.46 | 20.07 | 22.62 | 17.46 | 19.92 | 21.41 | 26.79 |

# Supplementary Table S2. Underlying osteoporotic sheep data points of Figure 3B.

| ***CF^D^_8min_*(N)** | **Distraction day** | | | | | | | | | | | | | | |
| --- | --- | --- | --- | --- | --- | --- | --- | --- | --- | --- | --- | --- | --- | --- | --- |
| **Sheep code** | **1** | **2** | **3** | **4** | **5** | **6** | **7** | **8** | **9** | **10** | **11** | **12** | **13** | **14** | **15** |
| 1 | - | - | 6.67 | 4.36 | 6.33 | 9.14 | - | -0.89 | 6.17 | - | - | - | 1.22 | - | 5.69 |
| 2 | - | 7.19 | 0.28 | - | - | 17.97 | - | - | - | - | - | - | 27.61 | - | 35.55 |
| 3 | 8.97 | 9.94 | 13.15 | 12.60 | 16.49 | 14.81 | 18.76 | 21.39 | 24.59 | 22.87 | 26.13 | 22.21 | 25.60 | 26.81 | 51.20 |
| 4 | 5.74 | 7.96 | 4.68 | 8.04 | 9.19 | 8.91 | 15.77 | 18.16 | 16.91 | 17.66 | 16.30 | 18.75 | 23.05 | 22.37 | 31.69 |
| 5 | 13.18 | 11.24 | 13.67 | 10.73 | 17.18 | 17.13 | 16.03 | 19.86 | 23.70 | 16.62 | 23.46 | 42.05 | 60.62 | 44.80 | - |
| 6 | 4.20 | 9.84 | 10.46 | 14.71 | 17.96 | 20.22 | 25.32 | 24.71 | 30.97 | 26.84 | 36.09 | 31.50 | 39.92 | 43.06 | 56.20 |
| 7 | - | 11.43 | 10.15 | 16.93 | 13.41 | 22.05 | 20.58 | 22.84 | 23.07 | 40.16 | 27.71 | 46.07 | 32.75 | 53.78 | 49.08 |
| 8 | 3.65 | 9.39 | - | 14.96 | 29.62 | 33.70 | 37.03 | 37.93 | 46.42 | 46.93 | 59.89 | 57.65 | 27.30 | 34.69 | 41.86 |
| 9 | 3.20 | 6.72 | 3.71 | 7.52 | 8.11 | 14.72 | 16.63 | 17.64 | 18.23 | 19.18 | 22.21 | 24.08 | 24.14 | 26.23 | 27.06 |
| 10 | - | 5.84 | - | 3.77 | 3.93 | 2.57 | 8.38 | 2.93 | 17.14 | 20.04 | 18.09 | - | 13.43 | 21.85 | 17.39 |
| Mean | 6.49 | 8.84 | 7.85 | 10.40 | 13.58 | 16.12 | 19.81 | 18.29 | 23.02 | 26.29 | 28.74 | 34.62 | 27.56 | 34.20 | 35.08 |
| Std | 3.89 | 2.00 | 4.78 | 4.76 | 7.85 | 8.52 | 8.45 | 11.53 | 11.14 | 11.26 | 13.99 | 14.40 | 15.64 | 11.86 | 16.59 |

# Supplementary Table S3. Underlying osteoporotic sheep data points of Figure 3C.

| ***CF^D^_20min_*(N)** | **Distraction day** | | | | | | | | | | | | | | |
| --- | --- | --- | --- | --- | --- | --- | --- | --- | --- | --- | --- | --- | --- | --- | --- |
| **Sheep code** | **1** | **2** | **3** | **4** | **5** | **6** | **7** | **8** | **9** | **10** | **11** | **12** | **13** | **14** | **15** |
| 1 | - | - | - | - | - | - | - | - | - | - | - | - | - | - | - |
| 2 | - | - | - | - | - | - | - | - | - | - | - | - | - | - | - |
| 3 | - | 8.23 | - | - | - | 12.08 | 13.93 | 17.23 | 18.43 | 17.32 | 18.90 | 16.33 | 19.16 | 20.54 | 47.35 |
| 4 | 4.48 | 6.71 | 3.54 | 6.97 | 6.92 | 7.20 | 13.49 | 14.65 | 13.61 | 12.47 | 12.76 | 14.43 | - | 19.52 | 30.95 |
| 5 | 11.30 | 10.47 | 10.60 | 6.29 | 16.36 | 12.08 | 12.61 | 20.73 | 21.60 | 14.13 | 22.12 | 35.11 | 58.66 | 39.36 | - |
| 6 | 2.73 | 9.15 | 9.59 | 13.66 | 13.60 | 17.06 | 21.26 | 21.50 | 26.13 | 22.65 | 32.98 | 24.01 | 34.08 | 39.26 | 54.67 |
| 7 | - | - | 7.32 | 17.92 | 11.06 | - | 19.39 | 24.17 | - | 38.61 | 28.85 | 35.70 | 30.25 | 47.36 | 44.08 |
| 8 | 3.42 | 5.97 | - | 12.44 | 20.32 | 23.65 | 23.46 | 26.18 | 32.94 | 32.96 | 44.91 | 47.79 | 12.21 | 22.67 | 23.46 |
| 9 | 2.19 | 6.45 | 3.88 | 6.15 | 7.42 | 12.93 | 14.68 | 14.17 | 14.94 | 15.63 | - | 18.84 | - | 20.22 | 21.09 |
| 10 | - | 2.25 | - | 2.33 | 4.08 | 2.46 | 5.52 | 0.86 | 14.40 | 14.63 | 13.53 | - | 10.13 | 16.43 | 10.32 |
| Mean | 4.82 | 7.03 | 6.99 | 9.39 | 11.39 | 12.49 | 15.54 | 17.44 | 20.29 | 21.05 | 24.86 | 27.46 | 27.42 | 28.17 | 33.13 |
| Std | 3.72 | 2.65 | 3.22 | 5.42 | 5.75 | 6.76 | 5.69 | 7.95 | 7.16 | 9.70 | 11.56 | 12.38 | 18.04 | 11.84 | 16.07 |

# Supplementary Table S4. Underlying osteoporotic sheep data points of Figure 3D.

| ***R* (%)** | **Time (min)** | | | | | | | | | |
| --- | --- | --- | --- | --- | --- | --- | --- | --- | --- | --- |
| **Sheep code** | **2** | **4** | **6** | **8** | **10** | **12** | **14** | **16** | **18** | **20** |
| 1 | 67.13 | 68.79 | 72.18 | 73.89 | 74.52 | 70.80 | 82.43 | 75.71 | 72.83 | 70.07 |
| 2 | 24.78 | 32.97 | 39.66 | 27.66 | 24.73 | 24.86 | 37.99 | 26.32 | 20.34 | 26.13 |
| 3 | 22.69 | 28.65 | 31.80 | 34.07 | 36.27 | 38.16 | 39.25 | 40.02 | 41.61 | 42.27 |
| 4 | 18.94 | 23.61 | 26.46 | 28.63 | 30.06 | 31.87 | 33.20 | 34.34 | 35.67 | 36.40 |
| 5 | 22.57 | 25.73 | 27.73 | 29.11 | 30.01 | 31.07 | 30.88 | 31.88 | 32.42 | 31.61 |
| 6 | 29.95 | 34.17 | 36.70 | 39.11 | 40.13 | 41.51 | 42.75 | 43.62 | 44.60 | 45.04 |
| 7 | 25.83 | 29.22 | 32.98 | 34.55 | 34.86 | 35.62 | 36.62 | 37.44 | 37.77 | 38.82 |
| 8 | 21.69 | 27.08 | 30.39 | 32.77 | 34.84 | 35.88 | 38.18 | 39.08 | 41.22 | 41.64 |
| 9 | 26.63 | 30.65 | 33.10 | 34.63 | 36.42 | 38.05 | 39.77 | 40.42 | 41.63 | 42.59 |
| 10 | 35.20 | 39.05 | 40.85 | 44.72 | 45.17 | 47.53 | 49.17 | 48.65 | 51.39 | 50.41 |
| Mean | 29.54 | 33.99 | 37.18 | 37.91 | 38.70 | 39.53 | 43.02 | 41.75 | 41.95 | 42.50 |
| Std | 13.98 | 13.01 | 13.16 | 13.64 | 13.80 | 12.58 | 14.72 | 13.44 | 13.60 | 11.86 |

# Supplementary Table S5. Underlying osteoporotic sheep data points of Figure 4A.

| ***K_cf_* (N/mm)** | **Distraction day** | | | | | | | | | | | | | | |
| --- | --- | --- | --- | --- | --- | --- | --- | --- | --- | --- | --- | --- | --- | --- | --- |
| **Sheep code** | **1** | **2** | **3** | **4** | **5** | **6** | **7** | **8** | **9** | **10** | **11** | **12** | **13** | **14** | **15** |
| 1 | - | - | 6.44 | 3.73 | 5.53 | 8.38 | - | 1.04 | 5.69 | - | - | - | 2.14 | - | 3.84 |
| 2 | - | 10.79 | 1.42 | - | - | 18.36 | - | - | - | - | - | - | 33.14 | - | 26.90 |
| 3 | 9.47 | 9.39 | 11.93 | 11.78 | 13.67 | 12.90 | 15.80 | 18.02 | 21.31 | 19.42 | 21.09 | 17.81 | 21.92 | 21.91 | 49.31 |
| 4 | 5.53 | 7.26 | 4.68 | 7.37 | 8.28 | 7.79 | 14.29 | 15.67 | 14.51 | 13.12 | 13.54 | 15.51 | 22.40 | 20.56 | 31.47 |
| 5 | 12.21 | 10.96 | 12.51 | 8.96 | 16.91 | 18.09 | 14.27 | 20.51 | 22.37 | 15.19 | 22.91 | 36.17 | 59.38 | 42.19 | - |
| 6 | 4.22 | 9.80 | 10.94 | 14.24 | 17.06 | 18.24 | 24.38 | 23.59 | 28.29 | 25.37 | 32.74 | 27.61 | 37.02 | 42.87 | 56.29 |
| 7 | 13.70 | 10.29 | 8.81 | 16.95 | 11.90 | 22.12 | 20.28 | 24.00 | 23.84 | 39.31 | 27.66 | 45.23 | 32.41 | 48.52 | 47.59 |
| 8 | 4.48 | 7.74 | 5.25 | 15.45 | 23.80 | 27.22 | 31.21 | 28.54 | 35.21 | 34.99 | 45.71 | 47.62 | 12.09 | 25.78 | 24.74 |
| 9 | 3.07 | 6.71 | 3.95 | 6.75 | 8.19 | 13.77 | 15.00 | 15.86 | 16.14 | 16.25 | 22.17 | 18.71 | 19.77 | 20.38 | 24.03 |
| 10 | 3.17 | 4.44 | 2.78 | 3.10 | 4.36 | 2.95 | 7.37 | 2.99 | 14.66 | 16.09 | 14.60 | 29.16 | 12.17 | 17.87 | 11.48 |
| Mean | 6.98 | 8.60 | 6.87 | 9.81 | 12.19 | 14.98 | 17.83 | 16.69 | 20.22 | 22.47 | 25.05 | 29.73 | 25.24 | 30.01 | 30.63 |
| Std | 4.22 | 2.20 | 3.95 | 5.05 | 6.32 | 7.29 | 7.32 | 9.30 | 8.66 | 9.84 | 10.44 | 12.38 | 16.14 | 12.36 | 17.58 |

# Supplementary Table S6. Underlying osteoporotic sheep data points of Figure 4B.

| ***K_gs_* (N/mm)** | **Distraction day** | | | | | | | | | | | | | | |
| --- | --- | --- | --- | --- | --- | --- | --- | --- | --- | --- | --- | --- | --- | --- | --- |
| **Sheep code** | **1** | **2** | **3** | **4** | **5** | **6** | **7** | **8** | **9** | **10** | **11** | **12** | **13** | **14** | **15** |
| 1 | - | - | 12.15 | 16.34 | 28.94 | 9.25 | - | 28.17 | 27.08 | - | - | - | 16.05 | - | 9.73 |
| 2 | - | 15.03 | 17.75 | - | - | 16.77 | - | - | - | - | - | - | 15.59 | - | 44.35 |
| 3 | 11.29 | 17.28 | 19.21 | 12.80 | 24.87 | 20.15 | 28.07 | 20.66 | 31.13 | 22.56 | 28.85 | 26.13 | 38.89 | 30.39 | 28.92 |
| 4 | 17.42 | 8.16 | 6.95 | 7.23 | 10.31 | 10.29 | 10.42 | 12.26 | 11.11 | 17.08 | 17.56 | 15.65 | 12.98 | 17.59 | 9.75 |
| 5 | 6.00 | 11.49 | 10.60 | 16.80 | 11.74 | 11.63 | 11.25 | 13.54 | 8.74 | 10.82 | 13.57 | 20.09 | 8.86 | 13.60 | - |
| 6 | 16.42 | 20.07 | 24.62 | 18.10 | 20.50 | 16.19 | 28.02 | 26.56 | 19.72 | 21.85 | 30.13 | 33.36 | 32.23 | 37.55 | 23.01 |
| 7 | 9.87 | 13.57 | 20.71 | 15.65 | 11.59 | 12.73 | 16.88 | 8.20 | 13.98 | 14.57 | 15.64 | 23.80 | 34.82 | 33.27 | 38.92 |
| 8 | 21.49 | 30.22 | 23.17 | 36.01 | 44.57 | 46.07 | 41.93 | 42.90 | 46.33 | 47.67 | 47.10 | 30.54 | 48.04 | 48.55 | 50.66 |
| 9 | 10.87 | 11.79 | 11.97 | 12.25 | 16.69 | 11.88 | 18.33 | 15.43 | 16.74 | 16.00 | 19.56 | 18.71 | 19.73 | 18.62 | 25.58 |
| 10 | 4.50 | 11.87 | 5.66 | 7.00 | 7.12 | 12.09 | 18.67 | 15.07 | 18.10 | 21.27 | 18.01 | 9.55 | 22.42 | 17.32 | 24.05 |
| Mean | 12.23 | 15.50 | 15.28 | 15.80 | 19.59 | 16.70 | 21.70 | 20.31 | 21.44 | 21.48 | 23.80 | 22.23 | 24.96 | 27.11 | 28.33 |
| Std | 5.82 | 6.54 | 6.71 | 8.57 | 11.81 | 10.84 | 10.49 | 10.72 | 11.75 | 11.33 | 11.15 | 7.85 | 12.83 | 12.30 | 14.20 |

# Supplementary Table S7. Underlying osteoporotic sheep data points of Figure 4C.

| ***η_gs_* (N·min/mm)** | **Distraction day** | | | | | | | | | | | | | | |
| --- | --- | --- | --- | --- | --- | --- | --- | --- | --- | --- | --- | --- | --- | --- | --- |
| **Sheep code** | **1** | **2** | **3** | **4** | **5** | **6** | **7** | **8** | **9** | **10** | **11** | **12** | **13** | **14** | **15** |
| 1 | - | - | 1.06 | 2.17 | 14.83 | 1.39 | - | 13.21 | 5.06 | - | - | - | 1.87 | - | 34.43 |
| 2 | - | 4.17 | 31.62 | - | - | 30.83 | - | - | - | - | - | - | 39.37 | - | 327.47 |
| 3 | 12.26 | 15.27 | 24.10 | 30.91 | 53.12 | 43.98 | 79.09 | 67.66 | 74.58 | 84.01 | 108.03 | 98.16 | 93.65 | 107.67 | 32.97 |
| 4 | 4.41 | 15.41 | 10.45 | 17.00 | 19.29 | 29.40 | 38.02 | 45.94 | 53.38 | 90.94 | 67.31 | 66.24 | 38.20 | 47.65 | 12.55 |
| 5 | 14.34 | 8.44 | 17.77 | 19.88 | 5.58 | 9.83 | 47.22 | 10.14 | 28.80 | 24.96 | 6.12 | 117.98 | 23.77 | 54.36 | - |
| 6 | 12.10 | 12.77 | 19.43 | 18.85 | 26.40 | 47.49 | 33.24 | 32.63 | 60.57 | 52.65 | 78.02 | 81.06 | 63.17 | 12.39 | 10.79 |
| 7 | 0.45 | 1.84 | 18.95 | 6.68 | 22.42 | 10.72 | 17.27 | 5.92 | 16.11 | 15.41 | 12.93 | 25.91 | 33.70 | 133.90 | 22.81 |
| 8 | 8.90 | 31.29 | 9.70 | 64.17 | 138.89 | 162.72 | 169.69 | 191.81 | 229.59 | 238.11 | 306.90 | 210.43 | 308.70 | 195.20 | 317.78 |
| 9 | 16.75 | 7.72 | 3.44 | 19.62 | 17.42 | 23.83 | 39.86 | 32.29 | 50.51 | 66.97 | 34.40 | 104.96 | 94.34 | 119.03 | 68.03 |
| 10 | 0.03 | 2.66 | 0.21 | 11.86 | 8.22 | 4.68 | 24.09 | 6.07 | 52.92 | 81.62 | 74.14 | 4.80 | 38.55 | 79.03 | 116.11 |
| Mean | 8.65 | 11.06 | 13.67 | 21.24 | 34.02 | 36.49 | 56.06 | 45.07 | 63.50 | 81.83 | 85.98 | 88.69 | 73.53 | 93.65 | 104.77 |
| Std | 6.35 | 9.15 | 10.43 | 18.13 | 41.67 | 47.11 | 49.51 | 58.79 | 66.11 | 68.90 | 95.78 | 62.76 | 87.61 | 57.63 | 127.77 |

# Supplementary Table S8. Underlying osteoporotic sheep data points of Figure 4D.

| ***τ_gs_* (min)** | **Distraction day** | | | | | | | | | | | | | | |
| --- | --- | --- | --- | --- | --- | --- | --- | --- | --- | --- | --- | --- | --- | --- | --- |
| **Sheep code** | **1** | **2** | **3** | **4** | **5** | **6** | **7** | **8** | **9** | **10** | **11** | **12** | **13** | **14** | **15** |
| 1 | - | - | 0.09 | 0.13 | 0.51 | 0.15 | - | 0.47 | 0.19 | - | - | - | 0.12 | - | 3.54 |
| 2 | - | 0.28 | 1.78 | - | - | 1.84 | - | - | - | - | - | - | 2.52 | - | 7.38 |
| 3 | 1.09 | 0.88 | 1.25 | 2.41 | 2.14 | 2.18 | 2.82 | 3.28 | 2.40 | 3.72 | 3.74 | 3.76 | 2.41 | 3.54 | 1.14 |
| 4 | 0.25 | 1.89 | 1.50 | 2.35 | 1.87 | 2.86 | 3.65 | 3.75 | 4.81 | 5.32 | 3.83 | 4.23 | 2.94 | 2.71 | 1.29 |
| 5 | 2.39 | 0.73 | 1.68 | 1.18 | 0.48 | 0.84 | 4.20 | 0.75 | 3.29 | 2.31 | 0.45 | 5.87 | 2.68 | 4.00 | - |
| 6 | 0.74 | 0.64 | 0.79 | 1.04 | 1.29 | 2.93 | 1.19 | 1.23 | 3.07 | 2.41 | 2.59 | 2.43 | 1.96 | 0.33 | 0.47 |
| 7 | 0.05 | 0.14 | 0.91 | 0.43 | 1.94 | 0.84 | 1.02 | 0.72 | 1.15 | 1.06 | 0.83 | 1.09 | 0.97 | 4.02 | 0.59 |
| 8 | 0.41 | 1.04 | 0.42 | 1.78 | 3.12 | 3.53 | 4.05 | 4.47 | 4.96 | 5.00 | 6.52 | 6.89 | 6.43 | 4.02 | 6.27 |
| 9 | 1.54 | 0.66 | 0.29 | 1.60 | 1.04 | 2.01 | 2.17 | 2.09 | 3.02 | 4.19 | 1.76 | 5.61 | 4.78 | 6.39 | 2.66 |
| 10 | 0.01 | 0.22 | 0.04 | 1.69 | 1.16 | 0.39 | 1.29 | 0.40 | 2.92 | 3.84 | 4.12 | 0.50 | 1.72 | 4.56 | 4.83 |
| Mean | 0.81 | 0.72 | 0.87 | 1.40 | 1.50 | 1.76 | 2.55 | 1.91 | 2.87 | 3.48 | 2.98 | 3.80 | 2.65 | 3.70 | 3.13 |
| Std | 0.83 | 0.53 | 0.66 | 0.79 | 0.85 | 1.16 | 1.32 | 1.56 | 1.53 | 1.45 | 1.99 | 2.31 | 1.81 | 1.72 | 2.55 |

# Supplementary Table S9. Osteoporotic sheep data points of Figure 5B.

| ***GRF/BW*** | **Day after surgery** | | |  |  |  |  |  |  |  |  |  |  |  |  |  |  |  |  |  |  |  |  |
| --- | --- | --- | --- | --- | --- | --- | --- | --- | --- | --- | --- | --- | --- | --- | --- | --- | --- | --- | --- | --- | --- | --- | --- |
| **Sheep code** | **7** | **9** | **11** | **12** | **13** | **14** | **15** | **17** | **18** | **19** | **20** | **21** | **22** | **24** | **25** | **26** | **27** | **28** | **29** | **30** | **31** | **32** | **33** |
| 1 | - | - | - | - | - | - | 0.134 | - | - | 0.135 | 0.161 | - | - | - | - | - | - | - | - | 0.161 | - | - | - |
| 3 | - | 0.245 | - | - | - | - | 0.336 | - | - | 0.245 | - | 0.289 | - | - | - | - | 0.293 | - | - | - | - | - | - |
| 6 | - | - | - | - | - | - | - | - | - | - | - | - | 0.061 | 0.062 | - | 0.062 | - | - | - | - | 0.075 | - | 0.089 |
| 7 | - | 0.172 | 0.112 | - | - | 0.199 | - | - | 0.174 | - | 0.140 | - | 0.111 | - | - | - | - | 0.074 | - | - | - | - | - |
| 8 | - | 0.235 | - | - | - | 0.263 | - | - | 0.180 | - | - | 0.224 | - | 0.187 | - | - | - | - | 0.274 | - | 0.253 | - | - |
| 9 | 0.455 | - | - | 0.322 | - | 0.313 | - | 0.280 | - | - | - | - | 0.134 | - | 0.117 | - | - | 0.228 | - | 0.200 | - | 0.243 | - |
| 10 | - | - | 0.238 | - | 0.271 | - | 0.294 | - | 0.312 | - | - | - | - | - | 0.337 | - | - | 0.306 | - | - | - | - | - |

| ***GRF/BW*** | **Day after surgery** | | |  |  |  |  |  |  |  |  |  |  |  |  |  |  |  |  |  |  |  |  |  |
| --- | --- | --- | --- | --- | --- | --- | --- | --- | --- | --- | --- | --- | --- | --- | --- | --- | --- | --- | --- | --- | --- | --- | --- | --- |
| **Sheep code** | **34** | **35** | **36** | **38** | **39** | **41** | **42** | **43** | **44** | **45** | **46** | **48** | **50** | **51** | **52** | **53** | **56** | **57** | **59** | **61** | **64** | **65** | **67** | **70** |
| 1 | 0.132 | - | - | - | - | - | - | - | 0.102 | - | 0.119 | - | - | - | - | - | - | - | - | - | - | - | 0.056 | 0.069 |
| 3 | - | - | - | - | 0.342 | - | - | - | - | 0.222 | - | - | - | 0.169 | - | 0.260 | - | 0.165 | 0.211 | 0.174 | - | 0.233 | 0.258 | - |
| 6 | - | 0.094 | - | - | - | 0.130 | - | 0.131 | - | - | - | 0.144 | - | 0.147 | - | - | - | 0.284 | - | - | - | 0.292 | - | - |
| 7 | 0.162 | - | 0.050 | - | - | - | 0.164 | - | - | 0.267 | - | - | 0.173 | - | 0.055 | - | 0.197 | - | 0.251 | - | 0.289 | - | - | 0.294 |
| 8 | - | 0.217 | - | 0.078 | - | - | 0.078 | - | - | - | - | - | - | - | - | - | - | - | - | - | - | - | - | - |
| 9 | 0.297 | - | 0.312 | - | 0.281 | - | 0.281 | - | 0.303 | - | - | - | - | - | - | - | - | - | - | - | - | - | - | - |
| 10 | 0.208 | - | 0.149 | - | - | - | - | - | - | - | - | - | - | - | - | - | - | - | - | - | - | - | - | - |

| ***GRF/BW*** | **Day after surgery** | | |  |  |  |  |  |  |  |  |  |  |  |
| --- | --- | --- | --- | --- | --- | --- | --- | --- | --- | --- | --- | --- | --- | --- |
| **Sheep code** | **73** | **74** | **75** | **76** | **77** | **80** | **81** | **84** | **87** | **91** | **92** | **94** | **98** | **100** |
| 1 | - | 0.052 | - | 0.033 | - | - | 0.033 | 0.105 | - | 0.048 | - | - | - | - |
| 3 | 0.270 | - | 0.267 | - | 0.286 | - | - | 0.349 | - | - | 0.365 | - | 0.343 | - |
| 6 | 0.287 | - | - | - | - | 0.347 | - | 0.373 | - | - | - | 0.400 | - | 0.404 |
| 7 | - | - | - | - | - | 0.261 | - | - | 0.162 | 0.132 | - | - | - | - |
| 8 | - | - | - | - | - | - | - | - | - | - | - | - | - | - |
| 9 | - | - | - | - | - | - | - | - | - | - | - | - | - | - |
| 10 | - | - | - | - | - | - | - | - | - | - | - | - | - | - |

# Supplementary Table S10. Osteoporotic sheep data points of Figure 5C.

| ***CF^C^/IF*** | **Day after surgery** | | |  |  |  |  |  |  |  |  |  |  |  |  |  |  |  |  |  |  |  |  |
| --- | --- | --- | --- | --- | --- | --- | --- | --- | --- | --- | --- | --- | --- | --- | --- | --- | --- | --- | --- | --- | --- | --- | --- |
| **Sheep code** | **17** | **22** | **25** | **26** | **27** | **28** | **29** | **30** | **31** | **32** | **33** | **34** | **35** | **36** | **38** | **39** | **41** | **42** | **43** | **44** | **45** | **46** | **48** |
| 1 | - | - | - | - | - | - | - | - | - | - | - | - | - | - | - | - | - | - | - | 0.359 | - | 0.220 | - |
| 3 | - | - | - | - | 0.067 | - | - | - | - | - | - | - | - | - | - | 0.290 | - | - | - | - | 0.280 | - | - |
| 6 | - | - | - | 0.235 | - | - | - | - | 0.255 | - | 0.498 | - | 0.511 | - | - | - | 0.740 | - | 0.823 | - | - | - | 0.876 |
| 7 | - | - | - | - | - | 0.141 | - | - | - | - | - | 0.420 | - | 0.258 | - | - | - | 0.653 | - | - | 0.807 | - | - |
| 8 | - | - | - | - | - | - | 0.230 | - | 0.227 | - | - | - | 0.259 | - | 0.285 | - | - | 0.243 | - | - | - | - | - |
| 9 | 0.115 | 0.288 | 0.100 | - | - | 0.308 | - | 0.269 | - | 0.348 | - | 0.506 | - | 0.498 | - | 0.565 | - | 0.614 | - | 0.646 | - | - | - |
| 10 | - | - | 0.0369 | - | - | 0.0645 | - | - | - | - | - | 0.2678 | - | 0.2945 | - | - | - | - | - | - | - | - | - |

| ***CF^C^/IF*** | **Day after surgery** | | |  |  |  |  |  |  |  |  |  |  |  |  |  |  |  |  |  |  |  |  |  |
| --- | --- | --- | --- | --- | --- | --- | --- | --- | --- | --- | --- | --- | --- | --- | --- | --- | --- | --- | --- | --- | --- | --- | --- | --- |
| **Sheep code** | **50** | **51** | **52** | **53** | **56** | **57** | **59** | **61** | **64** | **65** | **67** | **70** | **73** | **74** | **75** | **76** | **77** | **80** | **81** | **84** | **87** | **91** | **92** | **98** |
| 1 | - | - | - | - | - | - | - | - | - | - | 0.768 | 0.186 | - | 0.391 | - | 0.291 | - | - | 0.435 | 0.581 | - | 0.267 | - | - |
| 3 | - | 0.354 | - | 0.459 | - | 0.412 | 0.497 | 0.407 | - | 0.592 | 0.627 | - | 0.673 | - | 0.663 | - | 0.730 | - | - | 0.800 | - | - | 0.843 | 0.855 |
| 6 | - | 0.873 | - | - | - | 0.935 | - | - | - | 0.940 | - | - | 0.934 | - | - | - | - | 0.964 | - | 0.978 | - | - | - | - |
| 7 | 0.803 | - | 0.716 | - | 0.852 | - | 0.884 | - | 0.900 | - | - | 0.908 | - | - | - | - | - | 0.949 | - | - | 0.950 | - | - | - |
| 8 | - | - | - | - | - | - | - | - | - | - | - | - | - | - | - | - | - | - | - | - | - | - | - | - |
| 9 | - | - | - | - | - | - | - | - | - | - | - | - | - | - | - | - | - | - | - | - | - | - | - | - |
| 10 | - | - | - | - | - | - | - | - | - | - | - | - | - | - | - | - | - | - | - | - | - | - | - | - |

# Supplementary Table S11. Osteoporotic sheep data points of Figure 5D.

| ***K_C_*(kN/mm)** | **Day after surgery** | | |  |  |  |  |  |  |  |  |  |  |  |  |  |  |  |  |  |  |  |  |
| --- | --- | --- | --- | --- | --- | --- | --- | --- | --- | --- | --- | --- | --- | --- | --- | --- | --- | --- | --- | --- | --- | --- | --- |
| **Sheep code** | **17** | **22** | **25** | **26** | **27** | **28** | **29** | **30** | **31** | **32** | **33** | **34** | **35** | **36** | **38** | **39** | **41** | **42** | **43** | **44** | **45** | **46** | **48** |
| 1 | - | - | - | - | - | - | - | - | - | - | - | - | - | - | - | - | - | - | - | 0.367 | - | 0.185 | - |
| 3 | - | - | - | - | 0.047 | - | - | - | - | - | - | - | - | - | - | 0.267 | - | - | - | - | 0.254 | - | - |
| 6 | - | - | - | 0.202 | - | - | - | - | 0.224 | - | 0.651 | - | 0.685 | - | - | - | 1.866 | - | 3.053 | - | - | - | 4.620 |
| 7 | - | - | - | - | - | 0.108 | - | - | - | - | - | 0.475 | - | 0.228 | - | - | - | 1.232 | - | - | 2.735 | - | - |
| 8 | - | - | - | - | - | - | 0.195 | - | 0.192 | - | - | - | 0.230 | - | 0.261 | - | - | 0.210 | - | - | - | - | - |
| 9 | 0.085 | 0.265 | 0.073 | - | - | 0.291 | - | 0.241 | - | 0.350 | - | 0.670 | - | 0.651 | - | 0.850 | - | 1.044 | - | 1.197 | - | - | - |
| 10 | - | - | 0.0251 | - | - | 0.0452 | - | - | - | - | - | 0.2395 | - | 0.2734 | - | - | - | - | - | - | - | - | - |

| ***K_C_*(kN/mm)** | **Day after surgery** | | |  |  |  |  |  |  |  |  |  |  |  |  |  |  |  |  |  |  |  |  |  |
| --- | --- | --- | --- | --- | --- | --- | --- | --- | --- | --- | --- | --- | --- | --- | --- | --- | --- | --- | --- | --- | --- | --- | --- | --- |
| **Sheep code** | **50** | **51** | **52** | **53** | **56** | **57** | **59** | **61** | **64** | **65** | **67** | **70** | **73** | **74** | **75** | **76** | **77** | **80** | **81** | **84** | **87** | **91** | **92** | **98** |
| 1 | - | - | - | - | - | - | - | - | - | - | 2.170 | 0.150 | - | 0.421 | - | 0.269 | - | - | 0.505 | 0.910 | - | 0.238 | - | - |
| 3 | - | 0.359 | - | 0.555 | - | 0.459 | 0.647 | 0.449 | - | 0.952 | 1.099 | - | 1.346 | - | 1.290 | - | 1.771 | - | - | 2.618 | - | - | 3.517 | 3.875 |
| 6 | - | 4.506 | - | - | - | 9.448 | - | - | - | 10.212 | - | - | 9.318 | - | - | - | - | - | - | - | - | - | - | - |
| 7 | 2.671 | - | 1.647 | - | 3.768 | - | 5.012 | - | 5.875 | - | - | 6.456 | - | - | - | - | - | 12.103 | - | - | 12.511 | - | - | - |
| 8 | - | - | - | - | - | - | - | - | - | - | - | - | - | - | - | - | - | - | - | - | - | - | - | - |
| 9 | - | - | - | - | - | - | - | - | - | - | - | - | - | - | - | - | - | - | - | - | - | - | - | - |
| 10 | - | - | - | - | - | - | - | - | - | - | - | - | - | - | - | - | - | - | - | - | - | - | - | - |

# Supplementary Table S12. Underlying osteoporotic sheep with heterogeneous callus data points of Figure 6A.

| ***CF^D^_peak_* (N)** | **Distraction day** | | | | | | | | | | | | | | |
| --- | --- | --- | --- | --- | --- | --- | --- | --- | --- | --- | --- | --- | --- | --- | --- |
| **Sheep code** | **1** | **2** | **3** | **4** | **5** | **6** | **7** | **8** | **9** | **10** | **11** | **12** | **13** | **14** | **15** |
| 1 | - | - | 18.55 | 20.43 | 31.56 | 17.63 | - | 29.32 | 32.89 | - | - | - | 18.22 | - | 15.10 |
| 2 | - | 25.90 | 21.73 | - | - | 37.46 | - | - | - | - | - | - | 51.26 | - | 67.54 |
| 3 | 23.19 | 29.61 | 34.64 | 27.38 | 42.87 | 36.77 | 48.75 | 42.98 | 58.28 | 46.71 | 55.71 | 48.84 | 67.61 | 58.12 | 83.63 |
| 4 | 23.68 | 17.13 | 12.93 | 16.22 | 20.74 | 20.08 | 27.46 | 31.03 | 28.50 | 33.56 | 34.93 | 34.62 | 37.53 | 42.39 | 42.66 |
| 5 | 20.23 | 24.69 | 25.70 | 28.63 | 31.14 | 34.64 | 28.37 | 35.32 | 33.38 | 28.89 | 36.87 | 62.63 | 72.47 | 61.99 | - |
| Mean | 22.37 | 24.33 | 22.71 | 23.17 | 31.58 | 29.32 | 34.86 | 34.66 | 38.26 | 36.39 | 42.50 | 48.70 | 49.42 | 54.17 | 52.23 |
| Std | 1.87 | 5.24 | 8.14 | 5.87 | 9.04 | 9.64 | 12.04 | 6.09 | 13.52 | 9.24 | 11.48 | 14.01 | 22.25 | 10.38 | 29.95 |

# Supplementary Table S13. Underlying osteoporotic sheep with homogeneous callus data points of Figure 6A.

| ***CF^D^_peak_* (N)** | **Distraction day** | | | | | | | | | | | | | | |
| --- | --- | --- | --- | --- | --- | --- | --- | --- | --- | --- | --- | --- | --- | --- | --- |
| **Sheep code** | **1** | **2** | **3** | **4** | **5** | **6** | **7** | **8** | **9** | **10** | **11** | **12** | **13** | **14** | **15** |
| 6 | 22.90 | 31.02 | 39.29 | 35.90 | 41.77 | 38.32 | 58.27 | 55.73 | 53.35 | 53.76 | 69.98 | 69.01 | 77.05 | 88.19 | 85.97 |
| 7 | 23.53 | 23.98 | 32.80 | 32.94 | 26.17 | 36.91 | 40.12 | 32.10 | 42.09 | 55.54 | 45.28 | 73.28 | 75.34 | 90.99 | 94.85 |
| 8 | 28.31 | 44.28 | 31.66 | 57.19 | 76.00 | 81.45 | 81.27 | 79.39 | 90.61 | 92.34 | 101.11 | 82.72 | 66.82 | 82.59 | 83.77 |
| 9 | 15.84 | 19.31 | 16.04 | 21.11 | 27.64 | 28.49 | 37.05 | 34.77 | 36.54 | 35.83 | 46.36 | 41.58 | 43.89 | 43.34 | 55.12 |
| 10 | 7.81 | 17.26 | 9.36 | 11.22 | 12.72 | 15.33 | 28.97 | 20.05 | 36.40 | 41.51 | 36.24 | 41.96 | 38.49 | 39.10 | 39.47 |
| Mean | 19.68 | 27.17 | 25.83 | 31.67 | 36.86 | 40.10 | 49.14 | 44.41 | 51.80 | 55.80 | 59.79 | 61.71 | 60.32 | 68.84 | 71.84 |
| Std | 7.99 | 10.93 | 12.56 | 17.32 | 24.18 | 24.86 | 20.92 | 23.40 | 22.76 | 22.04 | 26.25 | 18.87 | 17.99 | 25.44 | 23.45 |

# Supplementary Table S14. Underlying osteoporotic sheep with heterogenous callus data points of Figure 6B.

| ***CF^D^_8min_* (N)** | **Distraction day** | | | | | | | | | | | | | | |
| --- | --- | --- | --- | --- | --- | --- | --- | --- | --- | --- | --- | --- | --- | --- | --- |
| **Sheep code** | **1** | **2** | **3** | **4** | **5** | **6** | **7** | **8** | **9** | **10** | **11** | **12** | **13** | **14** | **15** |
| 2 | - | - | 6.67 | 4.36 | 6.33 | 9.14 | - | -0.89 | 6.17 | - | - | - | 1.22 | - | 5.69 |
| 3 | - | 7.19 | 0.28 | - | - | 17.97 | - | - | - | - | - | - | 27.61 | - | 35.55 |
| 4 | 8.97 | 9.94 | 13.15 | 12.60 | 16.49 | 14.81 | 18.76 | 21.39 | 24.59 | 22.87 | 26.13 | 22.21 | 25.60 | 26.81 | 51.20 |
| 5 | 5.74 | 7.96 | 4.68 | 8.04 | 9.19 | 8.91 | 15.77 | 18.16 | 16.91 | 17.66 | 16.30 | 18.75 | 23.05 | 22.37 | 31.69 |
| 6 | 13.18 | 11.24 | 13.67 | 10.73 | 17.18 | 17.13 | 16.03 | 19.86 | 23.70 | 16.62 | 23.46 | 42.05 | 60.62 | 44.80 | - |
| Mean | 9.30 | 9.08 | 7.69 | 8.93 | 12.30 | 13.59 | 16.85 | 14.63 | 17.84 | 19.05 | 21.96 | 27.67 | 27.62 | 31.33 | 31.03 |
| Std | 3.73 | 1.85 | 5.71 | 3.58 | 5.38 | 4.33 | 1.66 | 10.43 | 8.50 | 3.35 | 5.08 | 12.57 | 21.28 | 11.88 | 18.89 |

# Supplementary Table S15. Underlying osteoporotic sheep with homogenous callus data points of Figure 6B.

| ***CF^D^_8min_* (N)** | **Distraction day** | | | | | | | | | | | | | | |
| --- | --- | --- | --- | --- | --- | --- | --- | --- | --- | --- | --- | --- | --- | --- | --- |
| **Sheep code** | **1** | **2** | **3** | **4** | **5** | **6** | **7** | **8** | **9** | **10** | **11** | **12** | **13** | **14** | **15** |
| 8 | 4.20 | 9.84 | 10.46 | 14.71 | 17.96 | 20.22 | 25.32 | 24.71 | 30.97 | 26.84 | 36.09 | 31.50 | 39.92 | 43.06 | 56.20 |
| 9 | - | 11.43 | 10.15 | 16.93 | 13.41 | 22.05 | 20.58 | 22.84 | 23.07 | 40.16 | 27.71 | 46.07 | 32.75 | 53.78 | 49.08 |
| 11 | 3.65 | 9.39 | - | 14.96 | 29.62 | 33.70 | 37.03 | 37.93 | 46.42 | 46.93 | 59.89 | 57.65 | 27.30 | 34.69 | 41.86 |
| 13 | 3.20 | 6.72 | 3.71 | 7.52 | 8.11 | 14.72 | 16.63 | 17.64 | 18.23 | 19.18 | 22.21 | 24.08 | 24.14 | 26.23 | 27.06 |
| 15 | - | 5.84 | - | 3.77 | 3.93 | 2.57 | 8.38 | 2.93 | 17.14 | 20.04 | 18.09 | - | 13.43 | 21.85 | 17.39 |
| Mean | 3.68 | 8.64 | 8.11 | 11.58 | 14.61 | 18.65 | 21.59 | 21.21 | 27.17 | 30.63 | 32.80 | 39.83 | 27.51 | 35.92 | 38.32 |
| Std | 0.50 | 2.31 | 3.81 | 5.64 | 9.93 | 11.34 | 10.64 | 12.66 | 12.07 | 12.39 | 16.58 | 14.99 | 9.89 | 12.88 | 15.91 |

# Supplementary Table S16. Underlying osteoporotic sheep with heterogenous callus data points of Figure 6C.

| ***CF^D^_20min_* (N)** | **Distraction day** | | | | | | | | | | | | | | |
| --- | --- | --- | --- | --- | --- | --- | --- | --- | --- | --- | --- | --- | --- | --- | --- |
| **Sheep code** | **1** | **2** | **3** | **4** | **5** | **6** | **7** | **8** | **9** | **10** | **11** | **12** | **13** | **14** | **15** |
| 2 | - | - | - | - | - | - | - | - | - | - | - | - | - | - | - |
| 3 | - | - | - | - | - | - | - | - | - | - | - | - | - | - | - |
| 4 | - | 8.23 | - | - | - | 12.08 | 13.93 | 17.23 | 18.43 | 17.32 | 18.90 | 16.33 | 19.16 | 20.54 | 47.35 |
| 5 | 4.48 | 6.71 | 3.54 | 6.97 | 6.92 | 7.20 | 13.49 | 14.65 | 13.61 | 12.47 | 12.76 | 14.43 | - | 19.52 | 30.95 |
| 6 | 11.30 | 10.47 | 10.60 | 6.29 | 16.36 | 12.08 | 12.61 | 20.73 | 21.60 | 14.13 | 22.12 | 35.11 | 58.66 | 39.36 | - |
| Mean | 7.89 | 8.47 | 7.07 | 6.63 | 11.64 | 10.45 | 13.34 | 17.54 | 17.88 | 14.64 | 17.93 | 21.96 | 38.91 | 26.47 | 39.15 |
| Std | 4.82 | 1.89 | 4.99 | 0.48 | 6.68 | 2.82 | 0.67 | 3.05 | 4.02 | 2.46 | 4.76 | 11.43 | 27.93 | 11.17 | 11.60 |

# Supplementary Table S17. Underlying osteoporotic sheep with homogenous callus data points of Figure 6C.

| ***CF^D^_20min_* (N)** | **Distraction day** | | | | | | | | | | | | | | |
| --- | --- | --- | --- | --- | --- | --- | --- | --- | --- | --- | --- | --- | --- | --- | --- |
| **Sheep code** | **1** | **2** | **3** | **4** | **5** | **6** | **7** | **8** | **9** | **10** | **11** | **12** | **13** | **14** | **15** |
| 8 | 2.73 | 9.15 | 9.59 | 13.66 | 13.60 | 17.06 | 21.26 | 21.50 | 26.13 | 22.65 | 32.98 | 24.01 | 34.08 | 39.26 | 54.67 |
| 9 | - | - | 7.32 | 17.92 | 11.06 | - | 19.39 | 24.17 | - | 38.61 | 28.85 | 35.70 | 30.25 | 47.36 | 44.08 |
| 11 | 3.42 | 5.97 | - | 12.44 | 20.32 | 23.65 | 23.46 | 26.18 | 32.94 | 32.96 | 44.91 | 47.79 | 12.21 | 22.67 | 23.46 |
| 13 | 2.19 | 6.45 | 3.88 | 6.15 | 7.42 | 12.93 | 14.68 | 14.17 | 14.94 | 15.63 | - | 18.84 | - | 20.22 | 21.09 |
| 15 | - | 2.25 | - | 2.33 | 4.08 | 2.46 | 5.52 | 0.86 | 14.40 | 14.63 | 13.53 | - | 10.13 | 16.43 | 10.32 |
| Mean | 2.78 | 5.96 | 6.93 | 10.50 | 11.30 | 14.03 | 16.86 | 17.38 | 22.10 | 24.90 | 30.07 | 31.59 | 21.67 | 29.19 | 30.72 |
| Std | 0.62 | 2.84 | 2.87 | 6.21 | 6.20 | 8.88 | 7.12 | 10.29 | 9.02 | 10.60 | 12.96 | 12.90 | 12.25 | 13.39 | 18.12 |

# Supplementary Table S18. Statistical analysis of Figure 3A-C.

| ***p-value* (osteoporotic vs non-osteoporotic)** | **Distraction day** | | | | | | | | | | | | | | |
| --- | --- | --- | --- | --- | --- | --- | --- | --- | --- | --- | --- | --- | --- | --- | --- |
| **Distraction force** | **1** | **2** | **3** | **4** | **5** | **6** | **7** | **8** | **9** | **10** | **11** | **12** | **13** | **14** | **15** |
| ***CF^D^_peak_*** | 0.6828 | 0.4376 | 0.1212 | 0.3301 | 0.5818 | 0.4848 | 0.4000 | **0.0496** | **0.0176** | **0.0401** | **0.0027** | **0.0295** | 0.1119 | 0.2788 | **0.0112** |
| ***CF^D^_8min_*** | 0.2619 | 0.8252 | - | 1.0000 | 0.9091 | 0.7576 | 1.0000 | 0.6070 | 0.1447 | 0.7546 | **0.0451** | 0.7879 | 0.7576 | 0.0889 | 0.0636 |
| ***CF^D^_20min_*** | - | - | - | - | - | - | - | - | 0.2667 | 0.5697 | 0.6667 | - | - | - | - |

Significance between groups evaluated using Mann-Whitney U test. Bold *p-values* means *p* < 0.05.

# Supplementary Table S19. Statistical analysis of Figure 3D.

| ***p-value* (osteoporotic vs non-osteoporotic)** | **Time (min)** | | | | | | | | | |
| --- | --- | --- | --- | --- | --- | --- | --- | --- | --- | --- |
| **Force relaxation** | **2** | **4** | **6** | **8** | **10** | **12** | **14** | **16** | **18** | **20** |
| ***R*** | **0.0136** | **0.0068** | **0.0110** | **0.0110** | **0.0110** | **0.0160** | 0.0559 | **0.0160** | **0.0160** | **0.0110** |

Significance between groups evaluated using Mann-Whitney U test. Bold *p-values* means *p* < 0.05.

# Supplementary Table S20. Statistical analysis of Figure 4A-D.

| ***p-value* (osteoporotic vs non-osteoporotic)** | **Distraction day** | | | | | | | | | | | | | | |
| --- | --- | --- | --- | --- | --- | --- | --- | --- | --- | --- | --- | --- | --- | --- | --- |
| **Viscoelastic  model parameters** | **1** | **2** | **3** | **4** | **5** | **6** | **7** | **8** | **9** | **10** | **11** | **12** | **13** | **14** | **15** |
| ***K_cf_*** | 0.0727 | 0.1898 | 0.1818 | 0.5035 | 0.4364 | 0.2727 | 0.7111 | 0.3277 | 0.2721 | 0.6943 | 0.6216 | 0.5697 | 0.6061 | 0.3758 | 0.2601 |
| ***K_gs_*** | 0.9333 | 0.8981 | 0.7576 | 0.1063 | 0.5818 | 0.1212 | 0.2667 | **0.0496** | **0.0360** | **0.0093** | 0.0653 | **0.0283** | 0.2727 | 0.1333 | **0.0336** |
| ***η_gs_*** | 0.8081 | 0.6993 | 1.0000 | 0.1063 | 0.3273 | 0.4848 | 0.7111 | 0.1447 | 0.7756 | 0.6126 | 0.9433 | 0.5697 | 0.6061 | 0.7758 | 0.3301 |
| ***τ_gs_*** | 0.4606 | 0.8981 | 0.9091 | 0.6042 | 1.0000 | 0.7576 | 0.7111 | 0.7756 | 0.0879 | **0.0140** | 0.3543 | 0.2141 | 0.6061 | 0.0848 | 0.8252 |

Significance between groups evaluated using Mann-Whitney U test. Bold *p-values* means *p* < 0.05.

# Supplementary Table S21. Statistical analysis of Figure 6A-C.

| ***p-value* (osteoporotic homog. callus  vs osteoporotic heteg. callus)** | **Distraction day** | | | | | | | | | | | | | | |
| --- | --- | --- | --- | --- | --- | --- | --- | --- | --- | --- | --- | --- | --- | --- | --- |
| **Distraction force** | **1** | **2** | **3** | **4** | **5** | **6** | **7** | **8** | **9** | **10** | **11** | **12** | **13** | **14** | **15** |
| ***CF^D^_peak_*** | 0.7857 | 0.9048 | 0.8413 | 0.4127 | 0.9048 | 0.5476 | 0.2500 | 0.7302 | 0.1905 | 0.1429 | 0.3929 | 0.3929 | 0.4206 | 0.5714 | 0.2857 |
| ***CF^D^_8min_*** | 0.1000 | 0.7302 | 1.0000 | 0.5556 | 0.9048 | 0.4206 | 0.3929 | 0.4127 | 0.4127 | 0.1429 | 0.3929 | 0.2286 | 0.8413 | 1.0000 | 0.7302 |
| ***CF^D^_20min_*** | 0.2000 | 0.2286 | 1.0000 | 0.8571 | 1.0000 | 0.3429 | 0.2500 | 0.7857 | 0.6286 | 0.1429 | 0.2286 | 0.2286 | 0.5333 | 1.0000 | 0.5714 |

Significance between groups evaluated using Mann-Whitney U test. Bold *p-values* means *p* < 0.05.
